# Compact spectroscopy of keV to MeV X-rays from a laser wakefield accelerator

**DOI:** 10.1038/s41598-021-93689-5

**Published:** 2021-07-13

**Authors:** A. Hannasch, A. Laso Garcia, M. LaBerge, R. Zgadzaj, A. Köhler, J. P. Couperus Cabadağ, O. Zarini, T. Kurz, A. Ferrari, M. Molodtsova, L. Naumann, T. E. Cowan, U. Schramm, A. Irman, M. C. Downer

**Affiliations:** 1 Department of Physics, The University of Texas at Austin, Austin, TX 78712-1081 USA; 2 The Helmholtz-Zentrum Dresden-Rossendorf, Institute for Radiation Physics, 01328 Dresden, Germany; 3 Technische Universität Dresden, 01069 Dresden, Germany

**Keywords:** Techniques and instrumentation, Plasma physics, Laser-produced plasmas, Plasma-based accelerators, Physics, X-rays

## Abstract

We reconstruct spectra of secondary X-rays from a tunable 250–350 MeV laser wakefield electron accelerator from single-shot X-ray depth-energy measurements in a compact (7.5 × 7.5 × 15 cm), modular X-ray calorimeter made of alternating layers of absorbing materials and imaging plates. X-rays range from few-keV betatron to few-MeV inverse Compton to > 100 MeV bremsstrahlung emission, and are characterized both individually and in mixtures. Geant4 simulations of energy deposition of single-energy X-rays in the stack generate an energy-vs-depth response matrix for a given stack configuration. An iterative reconstruction algorithm based on analytic models of betatron, inverse Compton and bremsstrahlung photon energy distributions then unfolds X-ray spectra, typically within a minute. We discuss uncertainties, limitations and extensions of both measurement and reconstruction methods.

## Introduction

Accelerator-based sources of bright, hard X-rays have enabled decades of advances in materials science^1^, medicine^2,3^, geology^4^, warm dense matter science^5^, radiography of high-*Z* materials^6^ and non-destructive testing in industry^7^. The radio-frequency electron accelerators that underlie these sources, however, are limited to accelerating gradients of $\sim 100$ MeV/m^8^. Consequently they are tens to hundreds of meters long, expensive to build and operate and challenging to access. Laser wakefield accelerators (LWFAs) powered by intense laser pulses interacting with a plasma^9,10^ offer tabletop complements to conventional accelerators, but require a unique set of diagnostics^11^. With accelerating gradients of $\sim 100$ GeV/m, LWFAs can accelerate electron bunches within several cm to energies $E_e$ approaching 10 GeV^12^, with bandwidth $\Delta E_e/E_e \sim \,$1–15% and charge $Q \sim 100$ s of pC. LWFAs are emerging as versatile small-laboratory sources of fs hard X-ray pulses^13^, with photon energies and peak brilliance rivaling those of their conventional synchrotron counterparts, and with a growing list of applications^14–16^.

LWFAs can generate three types of secondary X-rays: betatron radiation, inverse Compton scattered (ICS) radiation, and bremsstrahlung. Betatron radiation originates from transverse undulations of accelerating electrons in a wake’s focusing fields, and is a natural byproduct of the acceleration process^17–20^. A LWFA producing 250–350 MeV electrons emits betatron X-rays with a synchrotron-like spectrum, with critical energy $E_c \sim$ several keV^20^. Enhanced betatron radiation can be generated when additional interaction with the laser pulse occurs^21–23^ or in tailored density profiles^24,25^ resulting in larger oscillation amplitudes and higher critical X-ray energies, however these effects are not observed in the results presented here. ICS radiation results from backscatter of counter-propagating laser photons of energy $E_L$ from accelerated electrons of Lorentz factor $\gamma _e$, upshifting the photons to energy $E_x \sim 4 \gamma _e^2 E_L$^26–28^. Thus ICS of $E_L = 1.5$ eV photons from electron bunches with peak energy in the range $250< E_x < 350$ MeV ($490< \gamma _e < 685$) generates X-rays with spectral peaks in the range $1.5< E_x < 3$ MeV. Bremsstrahlung X-rays result from collisions, and associated acceleration, of relativistic electrons passing through a converter after the accelerator, producing broadband X-rays with photon energy up to $E_e$^29,30^. Secondary X-ray photons from LWFAs thus span an energy range from several keV to several hundred MeV, enabling a wide range of applications^13,14^, but requiring an unusually versatile spectrometer for source characterization^14^.

Currently multiple types of spectrometers are required to cover the photon energy range of X-rays from LWFAs. For $E_x \le 20$ keV, X-ray-sensitive charge-coupled devices (CCDs) operating as photon counters can build up a histogram of the spectrum of low-flux X-rays by measuring the charge that individual X-ray photons deposit in single pixels or pixel groups^31–34^. For $1 \le E_x \le 90$ keV, Ross filter pair arrays, which take advantage of the wide distribution of K-edge absorption energies across the periodic table, can analyze the spectral content of X-rays in a single shot^35,36^. For $90< E_x < 500$ keV, the sharp absorption sensitivity of K-edges is left behind, but broader differential transmission curves of high-*Z* materials still enable lower-resolution spectral analysis^37,38^. For $E_x > 1$ MeV, differential transmission detectors lose resolution quickly, and Compton scattering and $e^+ e^-$ pair production become the main processes for resolving X-ray photon energy^39,40^. X-rays of $E_x > 1$ MeV impinge on a converter, generating Compton electrons and/or $e^+ e^-$ pairs that are energy-analyzed in a magnetic spectrometer. The energy of the secondary Compton electrons and $e^+ e^-$ pairs is related straightforwardly to that of the incident X-rays, provided the converter is thin enough to avoid multiple scattering events. This converter thickness requirement limits signal-to-noise ratio, often necessitating averaging over multiple shots. To date, Compton/pair-production spectrometers have only measured broadband X-ray spectra. They have not yet measured peaked spectra, e.g. from linear ICS^41^.

Here, we spectrally characterize betatron, bremsstrahlung and ICS X-rays from a 250–350 MeV LWFA in a single shot, using a single, compact, inexpensive instrument: a modular calorimeter consisting of a stack of absorbers of varying *Z* and thickness, interlaced with imaging plates (IPs). The present measurements utilized a single fixed stack design to analyze an unprecedented 4-decade photon-energy range, demonstrating the spectrometer’s universality. However, the design is easily modified to enhance sensitivity and/or resolution within a narrower spectral range of interest. The current geometry can diagnose energies as low as $\sim 7$ keV, typical of betatron radiation, and as high as 100–500 MeV, typical of thick target bremsstrahlung radiation. We reconstruct spectra that are betatron-, ICS- or bremsstrahlung-dominated, as well as spectra containing a mixture of different types of X-rays with widely separated photon energies.

The calorimeter used here builds on designs developed by Jeon et al. for spectrally analyzing few-MeV betatron x-rays from a multi-GeV LWFA^42^, and by Garcia et al. for spectrally analyzing hard X-ray pulses from intense laser-solid interaction, natural X-ray emitters, and other sources^43^. Calorimeters consisting of alternating absorbers and detectors were also used in various other laser-plasma experiments^44–51^. The present study differs from prior work by demonstrating the universal applicability of calorimeter-based spectrometry to LWFA X-rays of all types—narrowband ICS as well as broadband betatron and bremsstrahlung emission—and to LWFAs of all sizes—tabletop MeV systems common in university laboratories as well as national-laboratory-scale multi-GeV systems studied by Jeon et al.^42^. We extend the work of Jeon et al.^42^ in four specific ways: (i) spectrometry of narrowband, tunable ICS X-rays; (ii) spectrometry of keV (instead of MeV) betatron X-rays, thereby enabling direct validation of reconstructed spectra against standard photon-counting CCD spectrometry^32,33,52^ for the first time; (iii) spectrometry of individual, as well as two and threefold mixtures of X-ray types, including mixtures of comparable vs. different intensity and overlapping vs. separated spectral content; (iv) establishment of the necessary requirements for the response matrix to accurately diagnose the relative contributions from bremsstrahlung when unfolding X-ray spectra of mixed origins. Such wide-ranging spectral analysis of ICS, betatron and bremsstrahlung X-rays from a sub-GeV LWFA, demonstrated here for the first time, is necessary to establish calorimetry as a standard, universal X-ray metrology for the global LWFA community. This analysis shows that a single compact, modular, home-built spectrometer can characterize all types of LWFA x-ray output accurately and efficiently.

## Results

### Generation and diagnosis of X-rays

**Figure 1:**
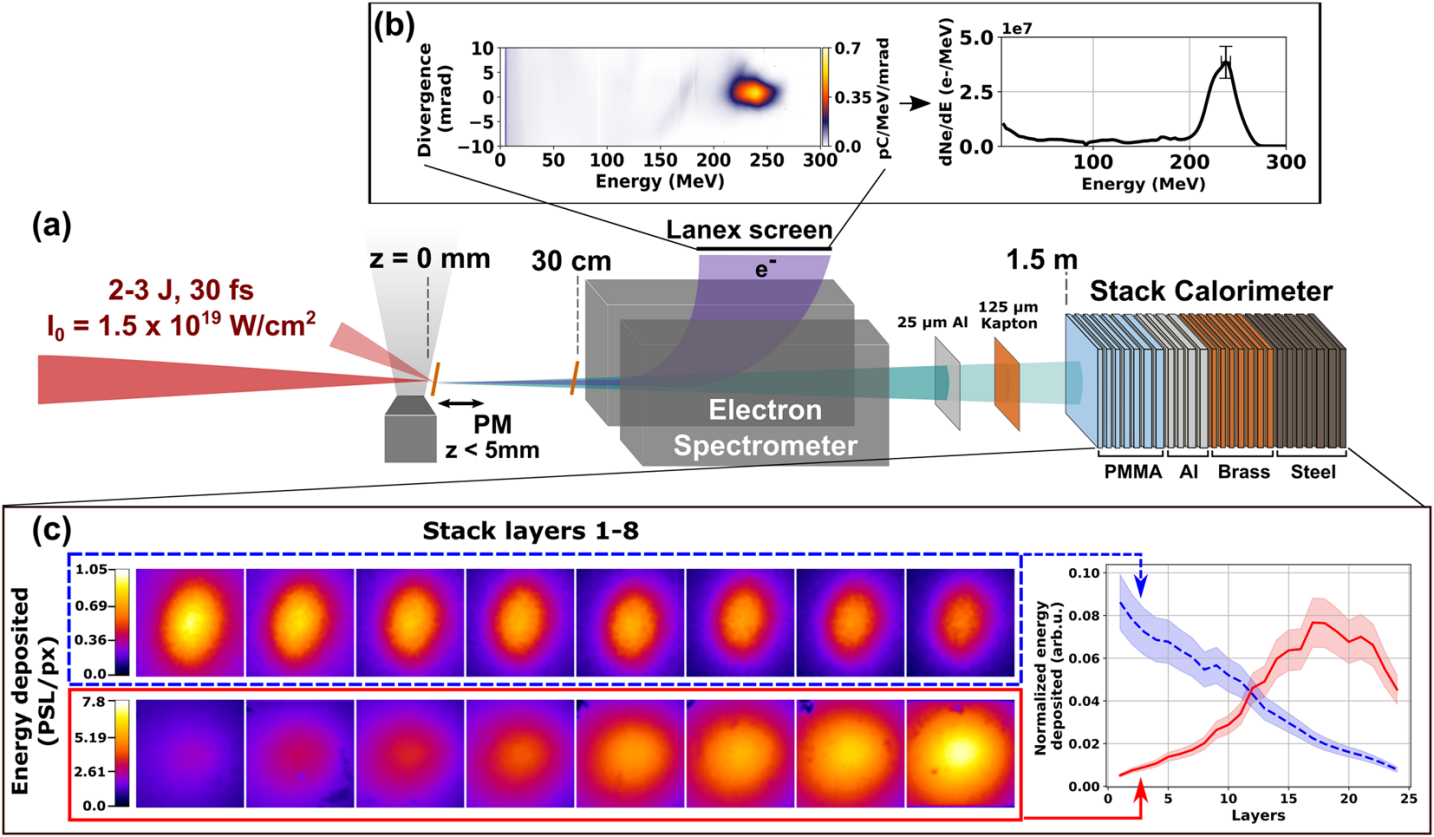
LWFA X-ray spectrometry overview. (**a**) Schematic set up showing (left to right) incident laser pulse, gas jet, tilted plasma mirror (PM) positioned at $0< z < 0.5$ cm from gas jet exit for generating ICS X-rays, converter at $z \approx 30$ cm for generating bremsstrahlung, 1 T magnetic electron spectrometer and X-ray stack calorimeter outside vacuum chamber. (**b**) Representative single-shot electron spectrum. Left: raw data from luminescent LANEX screen. Right: electron energy distribution integrated over emission angle. Error bars indicate uncertainties in electron energy (horizontal), due to uncertainty in electron entrance angle into magnetic spectrometer, and absolute charge (vertical), due to uncertainty in scintillator screen calibration, at the peak energy 230 MeV (see “Methods”/“Laser wakefield electron acceleration” for details). (**c**) Two depth-energy distributions from calorimeter. Top left (dashed blue box): first 8 image plate exposures for ICS-dominated radiation, generated with 25 μm-thick, low-*Z* Kapton PM at $z = 0.1$ cm. Bottom left (solid red box): same for bremsstrahlung-dominated radiation, generated with 800 μm-thick, high-*Z* tantalum converter at $z = 30$ cm. Color bars: relative scaling of deposited energy. Right: corresponding color-coded plots of transversely-integrated deposited energy (normalized to total deposited energy) vs. layer number for 24 layers. Shaded regions: calibration uncertainty (see Supplementary Material). Images in (**c**) were cropped to 16% of the total detector area to highlight differences between the two transverse energy deposition profiles, neither of which overfills the detector area.

Figure 1a presents a schematic overview of the LWFA X-ray spectrometry setup. A high-energy, ultra short laser pulse impinged on a nitrogen doped helium gas jet and excited a laser wakefield that accelerated electrons (see “Methods”). A magnetic electron spectrometer dispersed these electrons and diagnosed their energy distribution. Figure 1b shows an example of raw and angle integrated electron data for a 3-mm jet. A stack calorimeter consisting of 24 absorbing layers interspersed with IPs, recorded the depth-energy distribution of particle cascades initiated by secondary X-rays from the LWFA. Supplementary Table S1 lists absorber compositions and thicknesses and IP parameters for the stack used here. We generated and characterized four types of X-ray outputs:

1. *Pure betatron X-rays*. Betatron X-rays, generated in a 3-mm jet, propagated from LWFA exit ($z=0$) to calorimeter (entrance plane at $z=150$ cm), passing only through a 25 μm-thick Al laser blocking foil and a 125 μm-thick Kapton vacuum chamber window, both downstream of the *e*-spectrometer, which together blocked $<\,7$ keV X-rays. The *e*-beam generated no other X-rays outside the LWFA. We cross-checked unfolded betatron X-ray spectra in two ways: (a) by measuring betatron X-ray spectral histograms independently on separate, but similar, shots using a Pixis-XO 400BR photon-counting CCD sensitive to X-ray photon energies up to $\sim 30$ keV^34^; (b) by simulating the spectra generated by a single electron with various trial oscillation trajectories $r_\beta (t)$ using the classical radiation code CLARA^53^ (see Supplementary Material).
2. *Pure bremsstrahlung*. We used a 5-mm jet to maximize electron and photon energy, and inserted a thick, high-*Z* foil (e.g. 800 μm-thick Ta) at $z \sim \,30$ cm, which acted as a converter. Electrons entering the foil underwent collisions, generating forward bremsstrahlung. The foil blocked betatron X-rays completely.
3. *Bremsstrahlung + betatron X-rays*. We inserted a thin, low-*Z* foil (e.g. 25 μm-thick Kapton) at $z \sim \,30$ cm. It generated $\sim 400\times$ weaker bremsstrahlung, but transmitted most of the incident betatron X-rays. Thus the two had comparable flux at the detector.
4. *ICS X-rays*. We inserted the thin, low-*Z* foil (e.g. 25 μm-thick Kapton) at $0< z < 0.5$ cm. Here, the transmitted LWFA drive pulse was intense enough to ionize it, converting its front surface to an overdense plasma, or plasma mirror (PM), that retro-reflected the drive pulse back onto trailing electrons, generating ICS X-rays^16,37,54^. In this configuration, ICS X-rays dominated over betatron/bremsstrahlung background. Plasma mirroring, and thus ICS, were negligible for foils at $z = 30$ cm.

Figure 1c contrasts transverse energy profiles recorded by the first 8 IPs for an ICS-dominated (top left, blue dashed box) and a bremsstrahlung-dominated (bottom left, red solid box) shot. The plot on the right side of Fig. 1c shows transversely-integrated deposited energy vs. layer number for all 24 layers (see Table 5 in “Methods” for details on integration radius and total integrated energy for each source). These markedly different longitudinal energy profiles provide raw data for distinguishing the energy content of the two X-ray pulses.

**Figure 2:**
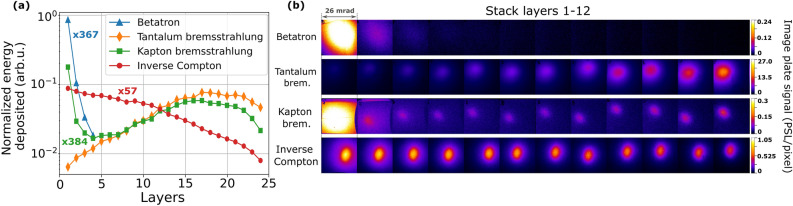
(**a**) Longitudinal profiles of deposited energy, generated by integrating the raw PSL/pixel data in (**b**) within the FWHM of each of the four LWFA X-ray outputs, are normalized to total energy deposited in the stack for each source. Scaling factors next to each curve indicate that the plotted energy deposition profile was multiplied by the indicated number to give its correct amplitude relative to tantalum bremsstrahlung X-rays (orange diamonds). Images in (**b**) were cropped to 64% of the total detector area.

Figure 2a compares normalized longitudinal energy profiles for the four X-ray outputs described above and the raw data from layers 1–12 is shown in Fig. 2b. Each data is multiplied by the factor shown in (a) to give its true amplitude relative to the pure bremsstrahlung source. Pure betatron X-rays (blue triangles, “Betatron”) deposit energy with progressively decreasing amplitude only in the first 4 layers, indicative of the short absorption depth of few-keV photons. The energy profile of mixed bremsstrahlung/betatron X-rays (green squares, “Kapton bremsstrahlung”) displays the same sharply-decaying betatron X-ray feature in the first few layers, but now augmented with broadly-distributed deposition deeper in the stack (peaking at layers 16–17) by higher-energy bremsstrahlung photons. Pure bremsstrahlung from a thick, high-*Z* foil (orange diamonds, “Tantalum bremsstrahlung”) generates no betatron feature in layers 1–4, only the characteristic broad “bremsstrahlung” peak in deeper layers, now stronger by a factor $\sim \,400$. ICS X-rays (red circles, “Inverse Compton”) deposit energy in a pattern distinct from the previous cases: energy deposition decreases monotonically throughout the stack. It is possible to recognize different classes of X-rays immediately from these “fingerprint” energy profiles alone, even before analyzing them to reveal their widely differing energy content quantitatively. The multiplicative factors illustrate the high dynamic range of the detector, which shows no saturation over a factor of nearly 500 in deposited energy. To the best of our knowledge, this is the first direct observation of the three different LWFA X-ray sources and their energy signatures from a single detector.

### Betatron X-rays

The betatron radiation spectrum is derived^17^ from Liénard-Wiechert potentials of accelerating electrons undergoing sinusoidal betatron oscillations of wavenumber $k_\beta = k_p/(2\gamma _e)^{1/2}$ and amplitude $r_\beta$ in the focusing fields of a plasma bubble. Here, $k_p$ is the plasma wavenumber. When the betatron strength parameter $a_\beta = \gamma _ek_\beta r_\beta$, analogous to a wiggler parameter, exceeds unity (for our experiments, $5 \le a_\beta \le 10$) and varies continuously during acceleration, radiation is generated in a forward-directed continuum of overlapping harmonics of the Doppler-upshifted betatron frequency $2\gamma _e^2ck_\beta /(1+a_\beta ^2/2)$ up to a critical frequency $\omega _c = 3\gamma _e^3k_\beta ^2cr_\beta$, beyond which intensity diminishes. The spectrum of radiation along the axis from a single electron then takes the form^17^

1 $$\begin{aligned} \frac{dN}{d(\hbar \omega )} = C \frac{\omega }{\omega _c^2} K_{2/3}^2\left( \frac{\omega }{\omega _c}\right) , \end{aligned}$$

where $C \approx 3 N_\beta e^2 \gamma _e^2 \Delta \Omega /(\hbar ^2 \pi ^2 \epsilon _0 c)$, $N_\beta$ is the number of betatron periods, $\Delta \Omega$ is an integrated solid angle and $K_{2/3}$ is a modified Bessel function. Here, we constrain the betatron photon spectrum to the form of Eq. (1), and use $\omega _c$ as a fit parameter.

**Figure 3:**
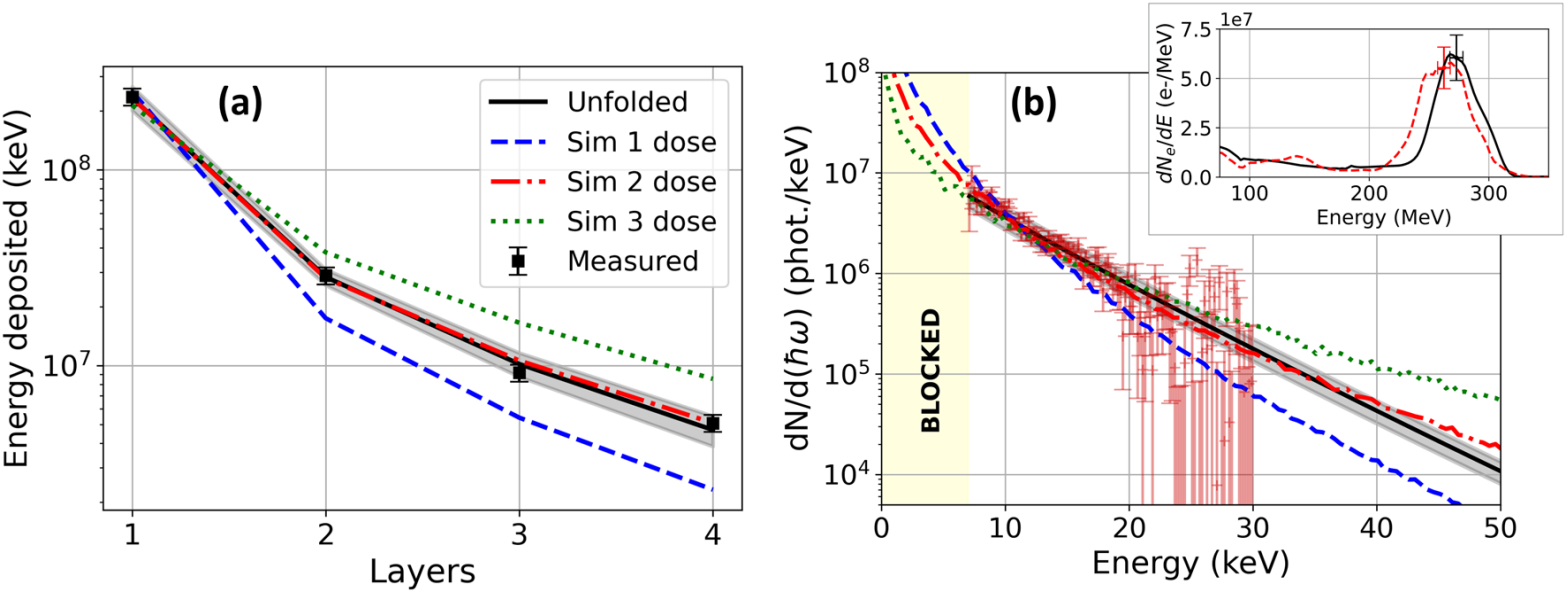
Betatron X-ray results. (**a**) Measured (black squares), unfolded (black solid curve) and simulated (colored curves labeled Sim 1, 2, 3) energy deposited in first 4 calorimeter stack layers. Error bars on data points are estimated from statistical variations in IP response; the corresponding uncertainty in unfolded energy deposition curve (grey shading) represents the standard deviation of an ensemble of unfolding calculations that reproduces this uncertainty in energy deposition (see “Methods”/“Error management” for details). (**b**) Corresponding unfolded spectrum (solid black curve) and its uncertainty (grey shading) of 20% determined from the energy calibration of the stack (see Supplementary materials) compared to betatron spectrum measured independently by X-ray photon counting (red data points). Colored curves: CLARA2 simulations of betatron X-ray spectra for *e*-trajectories $r_\beta (t)$ corresponding to final electron energy $E_e$ and oscillation amplitude $r_{\beta 0}$, respectively, of 280 MeV, $0.5\,\upmu$m (blue-dashed, Sim 1); 280 MeV, $0.9\,\upmu$m (red-dashed, Sim 2); 340 MeV, $0.9\,\upmu$m (green-dotted, Sim 3). Inset: electron spectra for calorimeter (black) and photon-counting (red) measurements.

Data points (squares) in Fig. 3a show a typical measured on-axis energy deposition profile $D_i^{(meas)}$ ($1 \le i \le 4$) from betatron X-rays generated by a 274 MeV ($\gamma _e = 536$) electron bunch with 18 MeV FWHM energy spread (see spectrum in inset of Fig. 3b, black curve) in $n_e = 5\times 10^{18}\,\text {cm}^{-3}$ plasma, compared to the unfolded energy distribution $D_i^{(calc)}$ [solid black curve in panel (a)]. We obtain best fit to the measured energy deposition with a X-ray photon spectrum $\frac{dN}{d(\hbar \omega )}(\hbar \omega ,\hbar \omega _c)$ of critical photon energy $\hbar \omega _c = 14 \pm 1.5$ keV, shown also by a solid black curve in the main panel of Fig. 3b. The number of photons within the FWHM of the betatron source is $5.5\pm 1.1\times 10^{7}$ over 7 keV. Yellow shading in Fig. 3b indicates energies that are blocked by the beam line elements and grey shading gives uncertainties in the unfolded energy profile (a) and spectrum (b), determined via the procedure described in “Methods”. From $E_c$, $n_e$, and $\gamma _e$, we estimate betatron radius $r_\beta = \omega _c/(3\gamma _e^3k_\beta ^2c) \approx 10^{23} \,E_c\,\mathrm{[keV]}/(\gamma _e^2\,n_e\,\mathrm{[cm^{-3}]}) = 1.0\pm 0.1~\upmu$m, or $a_\beta = 7 \pm 1$.

Red data points (+’s) in Fig. 3b show results of a typical independent X-ray spectral measurement using the photon-counting CCD, for a shot under the same conditions that yielded an electron bunch of nearly identical energy (Fig. 3b inset, red dashed curve). The X-ray spectrum is corrected for the transmission efficiency of the Al laser blocking foil, the Kapton vacuum chamber window (see Fig. 1a) and an additional filter that attenuated X-ray flux to less than one photon per pixel. The independently measured and unfolded spectra agree within combined uncertainties in the most sensitive range (8–20 keV) of the X-ray CCD.

The colored curves in Fig. 3b (blue dashed, red dot-dashed and green dotted curves) show X-ray spectra for three values of $r_\beta$ and $E_e$, selected from simulations for a range of $r_\beta$, $E_e$ values carried out using the classical radiation code CLARA2^53^ (see Supplementary Material). We chose the parameters for “Sim 2” (red dot-dashed curve), namely $r_\beta =0.9\pm 0.1$ μm and $E_e = 280\pm 20$ MeV (Table 1, second row from the bottom), to best match the unfolded and independently measured X-ray spectra over the sensitive range of the CCD detector. The stated uncertainties in $r_\beta$ and $E_e$ were generated from an ensemble of simulations, and represent variances from the best-fit values. Moreover, this simulated spectrum, when input into Eq. (8) using the same response matrix $R_{ij}$ used for the unfolding, yielded a calculated deposited energy (Fig. 3a, red dot-dashed curve, “Sim 2”) nearly indistinguishable from the measured (squares) and unfolded (solid black curve) energy deposition profiles. This good agreement corroborates the $r_\beta$ value inferred from the unfolding alone.

**Table 1:** Unfolded parameters for the betatron model based reconstruction (first row) and the simulated and corresponding unfolded parameters for the simulations labeled “Sim 1”, “Sim 2” and “Sim 3”.

	Electron parameters		Unfolded betatron parameters		
	$E_{pk}$ (MeV)	$r_\beta$ (μm)	$E_c$ (keV)	$r_\beta$ (μm)	$N_{phot}$
Unfolded	$274 \pm 18$	–	$14 \pm 1.5$	$1.0 \pm 0.1$	$5.5 \pm 1.1 \times 10^{7}$
Sim 1	280	0.5	9.9	0.66	–
Sim 2	$280 \pm 20$	$0.9 \pm 0.1$	$14 \pm 2$	$0.94 \pm 0.1$	–
Sim 3	340	0.9	19.4	0.87	–

The unfolded betatron parameters include the critical energy, betatron radius $r_\beta$ and number of photons with energy $> 7$ keV and within the FWHM of the beam. The simulations only provide the relative shape of the betatron spectra and do not include the photon number.

The parameters of the two additional CLARA2 simulation results shown in Fig. 3b, namely $r_\beta = 0.5$ μm, $E_e = 280$ MeV (blue dashed) and $r_\beta = 0.9$ μm, $E_e = 340$ MeV (green dotted) were chosen to bracket the sensitive range of this stack design. Both fall well outside the uncertainty range of the unfolded X-ray spectrum. Similarly their calculated energy distributions, shown by “Sim 1” (blue dashed) and “Sim 3” (green dotted), respectively, in Fig. 3a fall well outside the uncertainty range of the measured energy. When we ran the single-parameter unfolding algorithm on these calculated energy profiles, treated as synthetic data, we found $E_c = 10\pm 1$ keV and $r_\beta = 0.66\pm 0.2~\upmu \text{m}$ for “Sim 1” and a $E_c=19\pm 2$ keV and $r_\beta =0.85\pm 0.09$ for “Sim 3”, consistent with the original CLARA2 input parameters. These examples illustrate the degree to which the stack-based unfolding method can resolve betatron X-ray parameters associated with different acceleration conditions.

### Bremsstrahlung X-rays

Koch and Motz^55^ have compiled a comprehensive summary of cross-section approximations and experimental data for bremsstrahlung. Here we model bremsstrahlung spectra using either electron scattering cross-sections derived from the Born approximation or the so-called Kramers’ law. The Born differential cross-section for scattering of relativistic electrons to produce bremsstrahlung of photon energy $\hbar \omega$ has the analytic form (neglecting screening effects)^56^

2 $$\begin{aligned} \left( \frac{d\sigma }{d(\hbar \omega )}\right) _{Born} = \frac{16}{3} \frac{Z^2 r_e^2 \alpha }{\hbar \omega } \left( 1 - \frac{\hbar \omega }{E_0}+\frac{3\hbar ^2\omega ^2}{4 E_0^2}\right) \left[ \ln \left( \frac{2E_0(E_0-\hbar \omega )}{m_e c^2\hbar \omega }\right) -\frac{1}{2}\right] . \end{aligned}$$

Here, *Z* is the charge of the scattering nucleus, $\alpha$ the fine structure constant, $r_e$ the classical electron radius and $E_0$ the initial electron energy. Monoenergetic electrons passing through a thin ($L/L_0<<1$) low *Z* target (e.g. 25 μm-thick Kapton) lose negligible energy, so the bremsstrahlung spectrum, i.e. the number of photons per energy bin $dN/d(\hbar \omega )$, has the form of Eq. (2). Here, *L* is target thickness and $L_0$ the radiation length of the target material. Relativistic electrons ($E_0>>137mc^2Z^{-1/3}$) passing through a thick ($L/L_0\sim 1$), high *Z* target (e.g. 800 μm-thick Ta), on the other hand, experience energy-dependent alterations to the scattering cross-section because screening of the nucleus by atomic electrons becomes important in this limit, necessitating a correction to Eq. (2) (see Supplementary Material). We estimate $dN/d(\hbar \omega )$ by integrating the cross-section over target thickness, or equivalently over electron energy loss, assuming that electrons lose energy continuously to radiation at a rate $dE_0/dx = -E_0/L_0$.

3 $$\begin{aligned} \frac{dN}{d(\hbar \omega )} = n \int _{\hbar \omega }^{E_i} \frac{d\sigma }{d(\hbar \omega )} \frac{dE_0}{(-dE_0/dx)} = n L_0 \int _{\hbar \omega }^{E_i} \frac{1}{E_0} \frac{d\sigma }{d(\hbar \omega )} dE_0 \end{aligned}$$

The integration results in a piece-wise function, in which $dN/d(\hbar \omega )$ differs in form for $\hbar \omega$ greater than or less than the final electron energy $E_f$:

4a $$\begin{aligned} \left( \frac{dN}{d(\hbar \omega )}\right) _{low} = \frac{C}{\hbar \omega } \left( \ln {\frac{E_0}{E_f}}+\hbar \omega \left( \frac{1}{E_0}-\frac{1}{E_f}\right) - \frac{3}{8} (\hbar \omega )^2 \left( \frac{1}{E_0^2}-\frac{1}{E_f^2}\right) \right)  (\hbar \omega \ < E_f \le E_0) \end{aligned}$$

4b $$\begin{aligned} \left( \frac{dN}{d(\hbar \omega )}\right) _{high} = \frac{C}{\hbar \omega } \left( \ln {\frac{E_0}{\hbar \omega }}+\hbar \omega \left( \frac{1}{E_0}-\frac{1}{\hbar \omega }\right) - \frac{3}{8} (\hbar \omega )^2 \left( \frac{1}{E_0^2}-\frac{1}{(\hbar \omega )^2}\right) \right)  (E_f\le \hbar \omega \le E_0) \end{aligned}$$

where $C = 16 Z^2 r_e^2 \alpha n L_0/3$. The Born approximation model has been used widely to predict or model the properties of bremsstrahlung in experiments^55^.

Kramers derived the shape of the bremsstrahlung spectrum by a nonrelativistic semi-classical calculation that considered only continuous electron energy loss, but not discrete electron scattering events or radiation absorption^57^. Nevertheless, a common practice is to approximate the integration of the cross section over energy loss through a thick target using Kramers’ law, and to take radiation attenuation within the target into account using NIST attenuation data^58^. Moreover, since this integration is equivalent to integrating over incident electron energies, Kramers’ model is also widely used to describe bremsstrahlung from thin targets when there is electron energy spread. Kramers’ approximation for the bremsstrahlung spectrum has the analytic form

5 $$\begin{aligned} \frac{dN}{d(\hbar \omega )} \approx \frac{C}{\hbar \omega }(E_0 - \hbar \omega )\,, ~~~~~~~~~~ \hbar \omega \le E_0 \end{aligned}$$

where $C=8 \pi ^2 N_e r_e Z/(9 \sqrt{3} c \hbar )$. The symbol “$E_0$” in Eq. (5) actually represents the X-ray cutoff photon energy $E_{cutoff}$, but since this is close to the incident electron energy, we have approximated $E_{cutoff}\approx E_0$. When the incident electrons have a large energy spread, the same approximation holds, but now the symbol “$E_0$” denotes the *maximum* electron energy and an integral over the electron spectrum would need to be included in *C*. In practice, $E_0$ functions as an empirical parameter for fitting or unfolding spectra. Kramers’ Law has widely and successfully approximated observed bremsstrahlung spectra, even (its original assumptions notwithstanding) those generated by relativistic electrons in both thick and thin targets^55^.

**Figure 4:**
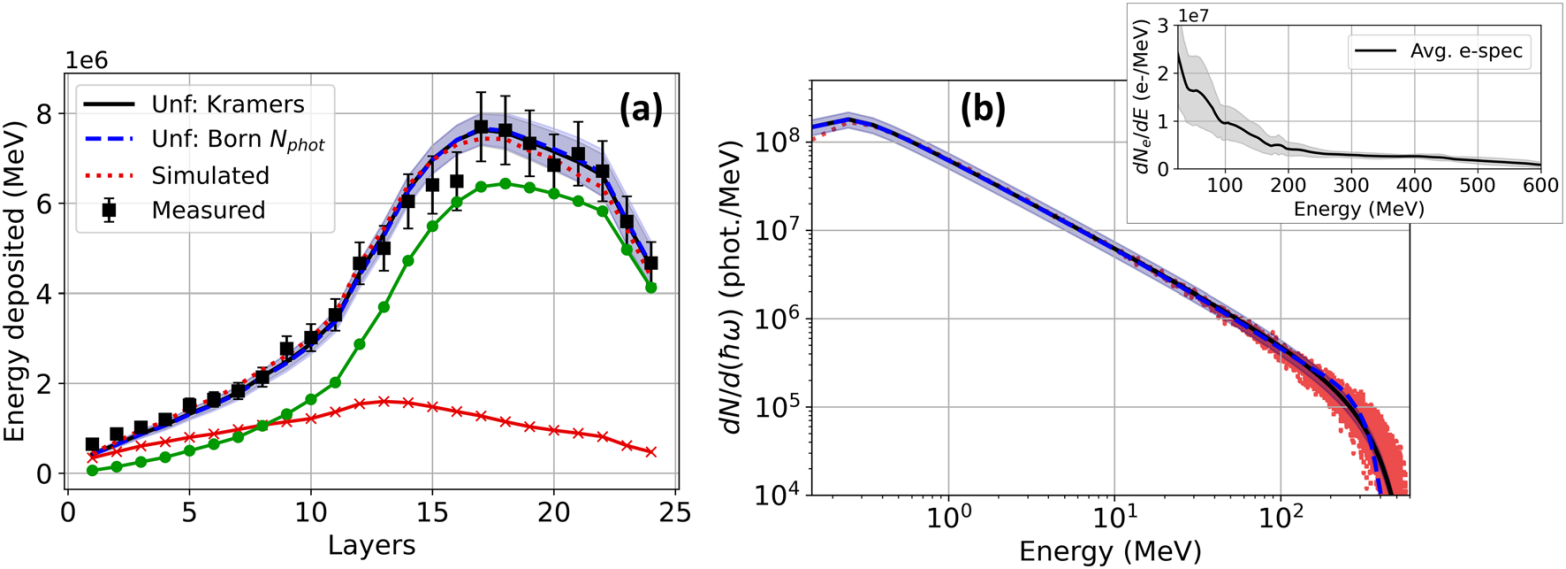
Bremsstrahlung X-ray results. (**a**) Comparison of measured (black squares), simulated (red dotted line) and unfolded energy deposition profiles based on Kramers’ law (black solid line) and the Born cross-section (blue dashed line) for the bremsstrahlung dominated case. The unfolded energy deposition profile based on Kramers’ law is further broken down into energy deposited by photons with energy $E_{ph} \le 35$ MeV (red $\times$’s) and photons with energy $E_{ph} > 35$ MeV (green circles). The corresponding unfolded and simulated spectrum are shown in (**b**) and the average electron spectrum for the previous 5 shots without the tantalum in the beam path in the inset of (**b**) with the standard deviation (shaded). The shaded regions in (**a**) represent the unfolding error and the shaded regions in (**b**) represent the 20% uncertainty in the absolute photon number as in Fig. 3.

Data points (black squares) in Fig. 4a show a typical energy deposition profile $D_i^{(meas)}$ $(1 \le i \le 24)$, integrated over the beam FWHM of 11.5 ± 0.4 mrad, from bremsstrahlung X-rays that LWFA electrons generated in an 800 μm-thick Ta target. The inset of Fig. 4b shows the energy distribution of the incident electrons, which had energy up to $\sim 500$ MeV, but large energy spread, as a result of emerging from an elongated 5 mm LWFA gas jet. Since the 800 μm-thick tantalum target significantly disrupted the electrons, preventing accurate on-shot measurement of their energy distribution, the black curve in this inset was obtained by averaging electron spectra of the 5 preceding shots *without* the tantalum in place, while the grey shaded region represents their standard deviation. The average spectrum corresponds to a total of $3.4 \pm 1.1 \times 10^9$ electrons and average bunch energy $160 \pm 30$ MeV, and was used for data analysis and Geant4 simulations.

Blue dashed and solid black curves in Fig. 4a show unfolded energy deposition profiles $D_i^{(calc)}$ for X-ray spectra based on the Born approximation (Eq. 4a,b) and Kramers’ law (Eq. 5), respectively. Figure 4b presents the corresponding best fit X-ray spectra with an average photon energy of $35\pm 4$ MeV ($36\pm 5$ MeV) for the unfolding based on Kramers’ law (the Born approximation). Photons with energy $E_{ph} \le 35$ MeV represent 76% of the bremsstrahlung photons and contribute 24% of the total deposited energy in the stack (Fig. 4a, red $\times$’s). Photons with energy $E_{ph} > 35$ MeV represent only 24% of the bremsstrahlung photons but contribute 76% of the total deposited energy in the stack (Fig. 4a, green circles). For this reason, it is essential for accurate unfolding of bremsstrahlung that the response matrix include photon energies up to the maximum value $E_0$, regardless of their photon number, since energetic photons contribute disproportionately to deposited energy. Red dotted curves in Fig. 4a,b show the simulated energy deposition profile and photon spectrum, respectively. Attenuation through the bremsstrahlung converter (e.g. 800 μm-thick Ta) is included in the unfolding algorithm and simulation resulting in the decreasing spectral intensity below 150 keV^58^. Unfolded and simulated energy deposition profiles are nearly indistinguishable from one another and fall within the 10% relative uncertainty of the unfolding over the full range of the stack. Unfolded and simulated spectra similarly agree, with only small differences at the high energy limit (< 20%) between the two models. Table 2 compares the bremsstrahlung beam parameters unfolded from the two models and obtained from the simulated spectrum. The uncertainty in the number of photons in the simulated beam reflects the uncertainty in the number of electrons incident on the Ta target. Moreover, electrons with energy $E_e \ge 200$ MeV represented 28% of the total charge but generated 83% of the total photons, 90% of the deposited energy in the stack and 93% of the total radiated energy in the resulting unfolded bremsstrahlung beam. Table 2 includes the photon efficiency $N_{ph}/N_{e}$ and energy efficiency $E_{rad}/E_{bunch}$ of the bremsstrahlung source considering only electrons with energy $E_e \ge 200$ MeV.

**Table 2:** Unfolded parameters for the two bremsstrahlung models and the simulated case including the average energy, cutoff energy, photon number, photon conversion efficiency $N_{ph}/N_e$ and energy conversion efficiency $E_{rad}/E_{bunch}$ from electrons with energy $E_e \ge 200$ MeV and photons over 100 keV within the FWHM of the bremsstrahlung transverse energy profile.

	$E_{avg}$ (MeV)	$E_{cutoff}$ (MeV)	$N_{phot}$	$N_{ph}/N_e$	$E_{rad}/E_{bunch}$
Simulated	36	$\sim 500$	$4.7 \pm 1.5 \times 10^{8}$	$0.41 \pm 0.13$	$0.046 \pm 0.015$
Unfolded: Kramers	$35 \pm 4$	$490 \pm 80$	$4.2 \pm 0.8 \times 10^{8}$	$0.37 \pm 0.07$	$0.040 \pm 0.008$
Unfolded: Born $N_{phot}$	$36 \pm 5$	$370 \pm 60$	$4.1 \pm 0.8 \times 10^{8}$	$0.36 \pm 0.07$	$0.040 \pm 0.008$

### Betatron + bremsstrahlung X-rays

**Figure 5:**
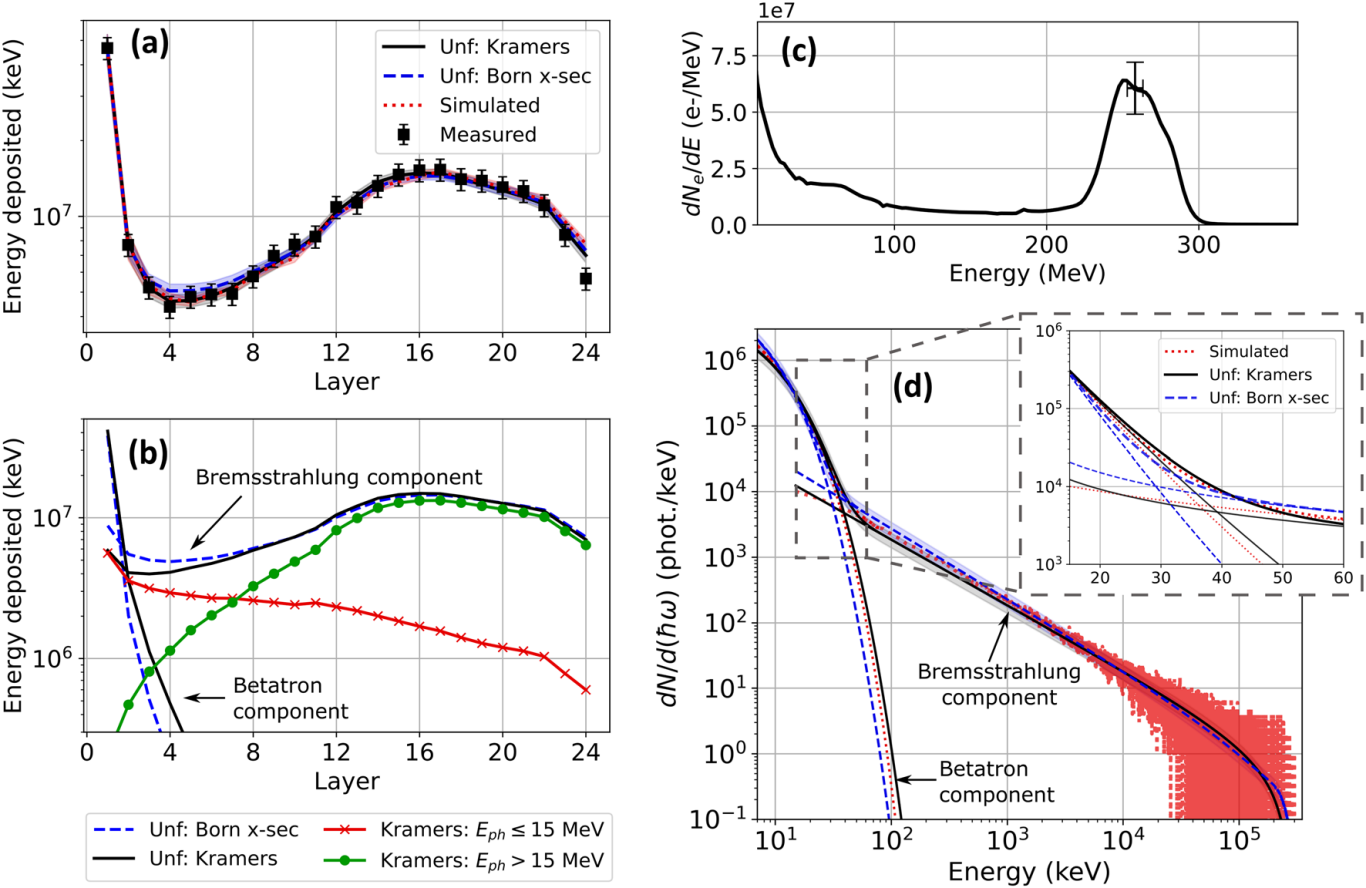
Combined betatron/bremsstrahlung X-ray results. (**a**) Measured energy deposition profile (black squares), unfolded profiles based on the betatron radiation model (Eq. 1) plus Kramers’ law (black solid) and Born (blue dashed) bremsstrahlung models, and Geant4-simulated profile (red dashed); (**b**) The same unfolded energy deposition profiles separated into betatron components assuming the Kramers’ law (black solid) and Born (blue dashed) bremsstrahlung models for the accompanying bremsstrahlung contribution. The unfolded bremsstrahlung energy deposition based on Kramers’ law is further broken down into energy deposited by photons with energy $E_{ph} \le 15$ MeV (red $\times$’s) and by photons with energy $E_{ph} > 15$ MeV (green circles); (**c**) the measured electron spectrum with error bars placed at the average electron energy in the quasi-monoenergetic peak and (**d**) the combined betatron and bremsstrahlung spectra scaled to the bremsstrahlung FWHM and extracted from unfolded and simulated profiles in (**a**). Again, the betatron spectral components are unfolded based on the Kramers’ law (black solid) and Born (blue dashed) models used to unfold the accompanying bremsstrahlung contributions; Inset: combined photon spectra from 15 to 60 keV where the dominant contribution changes from betatron to bremsstrahlung. Shaded regions denote unfolding error in (**a**), and uncertainty in absolute photon number in (**d**), as in Fig. 3.

The 25 μm-thick Kapton target was thick enough to generate detectable bremsstrahlung, yet thin enough to transmit most betatron radiation while negligibly perturbing the transverse spatial profile and energy spectrum of incident electrons. Data points (black squares) in Fig. 5a show a typical measured energy deposition profile $D_i^{(meas)}$ ($1 \le i \le 24$) using this target.

Betatron radiation deposited most of the energy in layer 1, bremsstrahlung most of the energy in layers 3–24, while the two sources deposited comparable energy in intermediate layer 2 as is illustrated by Figs. 2b and 5b. Because betatron and bremsstrahlung energy deposition profiles overlapped minimally, we analyzed and simulated each separately using models described in the previous two sections. We then unfolded the complete profile in one shot with the help of a single additional parameter describing their overall relative amplitude. For data in Fig. 5a, electrons originated from a 3-mm-long LWFA gas jet, yielding the energy spectrum with quasi-monoenergetic peak at $258\pm 16$ MeV shown in Fig. 5c, which we measured on the same shot as the X-ray energy deposition profile.

Black solid (blue dashed) curves in Fig. 5a represent unfolded deposited energy profiles based on Eq. (1) for betatron radiation, on Kramers’ Law (Born cross-section) for bremsstrahlung, and on an overall betatron/bremsstrahlung amplitude ratio parameter. We gave the Born cross-section model the form of Eq. (2) (rather than 3), since electrons lose negligible energy in the thin target. Both fitted curves fall within experimental error bars throughout the detector stack. We obtained the best fit to the energy deposition profiles with a *betatron* critical energy $E_c$ of $12 \pm 2$ keV ($9 \pm 2$ keV) and an average *bremsstrahlung* photon energy $E_{avg}$ of $15\pm 2$ MeV ($12\pm 3$ MeV) for the unfolding based on Kramers’ law (the Born approximation). Photons with energy $E_{ph} \le 15$ MeV represent 81% of the bremsstrahlung photons and contribute 24% of the total deposited energy in the stack (Fig. 5b, red $\times$’s) while photons with energy $E_{ph} > 15$ MeV represent 19% of the bremsstrahlung photons and contribute 76% of the total deposited energy in the stack (Fig. 5b, green circles). The relative importance of these high energy photons will be elaborated on in the “Discussion”. Figure 5d shows the corresponding betatron and bremsstrahlung spectra scaled to the energy within the FWHM of the bremsstrahlung beam, while the last two rows of Table 3 list unfolded model parameters for betatron radiation and bremsstrahlung within each beam’s respective FWHM. The $\sim 30\%$ difference in betatron parameters $E_c$ and $N_{phot}$ result from compensating for the difference between the two bremsstrahlung models in layers $1< i < 6$ as is illustrated in Fig. 5b.

**Table 3:** Betatron and bremsstrahlung X-ray parameters resulting from two model-based reconstructions of the combined energy deposition profile in the calorimeter, and from Geant4 simulation of the bremsstrahlung component.

	Betatron parameters		Bremsstrahlung parameters	
	$E_c$ (keV)	$N_{phot}$	$E_{avg}$ (MeV)	$N_{phot}$
Simulated	$11 \pm 3$	$4.4 \pm 0.9 \times 10^{7}$	16.0	$1.9 \times 10^{6}$
Unfolded: Kramers	$12 \pm 3$	$4.0 \pm 0.8 \times 10^{7}$	$15 \pm 2$	$1.7 \pm 0.3 \times 10^{6}$
Unfolded: Born x-sec	$9 \pm 2$	$5.1 \pm 1.1 \times 10^{7}$	$13 \pm 2$	$2.0 \pm 0.4 \times 10^{6}$

Betatron parameters include critical energy $E_c$, number of photons $N_{phot}$ with energy $> 7$ keV within the betatron FWHM. Bremsstrahlung parameters include average energy $E_{avg}$ and number of photons within the bremsstrahlung FWHM of the recorded calorimeter signal.

The red dotted line in Fig. 5a represents the “simulated” energy deposition profile. To obtain this curve, we first generated the bremsstrahlung part of the energy deposition profile in Geant4 using the measured electron spectrum (Fig. 5c), and scaled it vertically to match the measured energy deposition $D_i^{(meas)}$ in layers 8 through 24. We then used the remaining energy in the stack to unfold the betatron contribution based on Eq. (1). The simulated profile also falls within experimental error bars throughout the stack, and nearly overlaps the unfolded “Kramers” profile. Likewise, the corresponding simulated spectra (red dotted curves in Fig. 5d) and model parameters (Table 3, third row from bottom) closely match their unfolded “Kramers” model counterparts. Within uncertainty, we observed the same number $N_{phot}$ of betatron photons as from the pure betatron source. On the other hand, we observe 300 times fewer bremsstrahlung photons per electron from the thin Kapton target (Table 3) than from the thick tantalum target (Table 2).

### ICS X-rays

The ICS radiation spectrum is derived^28^ from Liénard-Wiechert potentials of accelerating electrons undergoing sinusoidal undulations in the electric field of a counter-propagating laser pulse. When the laser strength parameter $a_0 = 0.85 \lambda _0 (\upmu \text {m}) \sqrt{I_0 (10^{18}~ \text {W/cm}^2)}$, analogous to a wiggler parameter, is much less than unity, radiation is generated in a forward directed cone at the Doppler-upshifted fundamental frequency^28^ $4\gamma _e^2 \omega _0 /(1+a_0^2/2+\theta ^2\gamma _e^2)$. Here, $\omega _0$ is the central laser frequency (and $\hbar \omega _0$ = 1.55 eV the central photon energy) and $\theta$ the angle of observation relative to the electron propagation direction. Assuming $a_0 \ll 1$ and given an electron spectrum $N_e f(\gamma )$, the energy radiated per unit $\hbar \omega$ can be calculated:

6 $$\begin{aligned} \begin{aligned} \frac{d\mathcal {E}_x}{d(\hbar \omega )}&= 2 \pi \alpha N_e N_0^2 a_0^2\int _0^{\theta _{max}} \sin \theta d\theta \int d\gamma f(\gamma ) \gamma ^2 \left( \frac{1 + \gamma ^4 \theta ^4 }{\left( 1 + \gamma ^2 \theta ^2 \right) ^4}\right) Res(k,k_0) \end{aligned} \end{aligned}$$

Here, $Res(k,k_0)$ is sharply peaked at the resonant frequency. This integration can take additional time and requires knowledge of the electron spectrum $N_e f(\gamma )$. For a peaked electron spectrum with relative energy spread $\sigma _\gamma /\gamma _e \approx 0.1$, the angle-integrated ICS spectrum can be approximated by a Gaussian function with mean photon energy $E_x$ and variance $\sigma _{E_x}$ (see Supplementary Material):

7 $$\begin{aligned} \frac{d\mathcal {E}_x}{d(\hbar \omega )} \propto \hbar \omega \frac{dN}{d(\hbar \omega )} \propto C\exp \left( -\frac{(\hbar \omega -E_x)^2}{2 \sigma _{E_x}^2}\right) . \end{aligned}$$

Here, we express the spectral amplitude in terms of integrated X-ray pulse energy $\mathcal {E}_x = N\hbar \omega$ in order to retain a pure Gaussian function on the right-hand side. The parameters $E_x$ and $\sigma _{E_x}$ must satisfy two physical constraints: (*i*) $E_x$ cannot exceed $4 \gamma _{e}^2 \hbar \omega _0$; (*ii*) $\sigma _{E_x}/E_x$ must exceed the relative energy spread of the electron bunch, i.e. $\sigma _{E_x}/E_x > \sigma _\gamma /\gamma$. The values of $E_x$ and $\sigma _{E_x}$ extracted from data analysis can then help to diagnose a variety of physical effects involved in ICS with a plasma mirror, e.g. redshift of laser photon frequency $\omega _0$ during LWFA, which decreases $E_x$; laser frequency broadening (here, $\sigma _{E_L}/E_L \approx 0.1$ or larger), electron energy spread (here, $\sigma _\gamma /\gamma \approx .065$) and angular divergence (here, $\sigma _\theta \approx 1/\gamma _e$), and non-linear scattering (generation of harmonics)^59,60^, all of which contribute in quadrature to $\sigma _{E_x}$. Given the values of $\sigma _{E_L}/E_L$, $\sigma _\gamma /\gamma$ and $\sigma _\theta$ cited above, we constrain $\sigma _{E_x}/E_x$ to a practical lower bound of 0.35 during unfolding.

To generate ICS radiation, we used the thin Kapton foil to minimize bremsstrahlung, and placed it only $z = 0.1$ cm from the LWFA exit to ensure strong retro-reflection of the spent LWFA drive pulse via plasma mirroring, thereby maximizing ICS. Nevertheless betatron radiation from the LWFA leaked through the foil, and electrons from the LWFA generated some bremsstrahlung on passing through it. To remove the bremsstrahlung and betatron components, we took advantage of our ability, demonstrated in the preceding sections, to simulate the bremsstrahlung and betatron energy deposition profiles quantitatively and scale them to each shot based on the independently measured electron charge and average energy. We then subtracted this from the measured profile, leaving us with a pure ICS profile *only*. The ratio of energy in the scaled bremsstrahlung/betatron profile to the total measured energy is $\sim 11\%$ for shot 1 and $\sim 12\%$ for shot 2 which agrees with independent scintillator based measurements of the relative contributions^61^. The uncertainty in the final background subtracted ICS energy deposition profile incorporates the combined uncertainty in the measured data (10% relative uncertainty) and the scaled bremsstrahlung/betatron uncertainty which we estimate has an increased relative uncertainty of 15%. Thus, the final relative uncertainty is not constant for all layers and is higher for layers most affected by the subtraction procedure (layers 1 and 10–20). To include this modified uncertainty, the least squares optimization includes the *relative uncertainty* as a weighting for the unfolding. We will hereafter refer to the scaled bremsstrahlung/betatron profile as the background and the final ICS energy deposition profile after the subtraction procedure as the background-subtracted ICS data.

**Figure 6:**
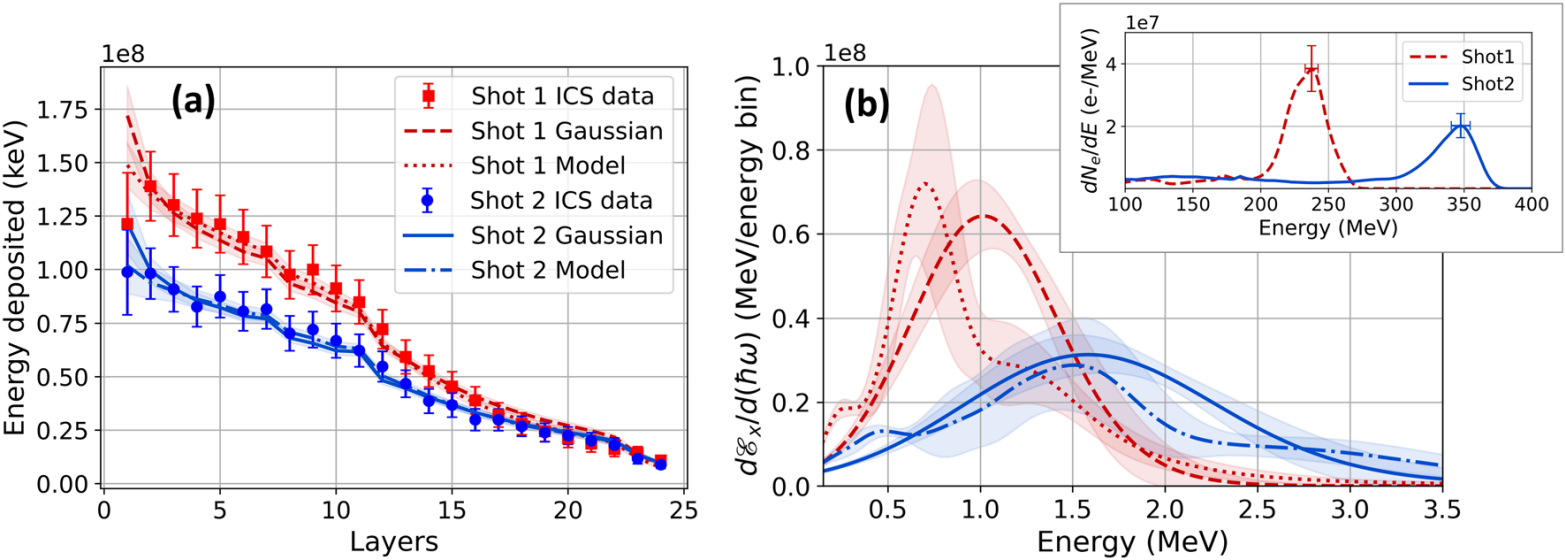
ICS X-ray results. (**a**) Data points: Background-subtracted ICS energy deposition profiles for two separate shots with peak electron energies $E_e = 236$ MeV (red squares) and 345 MeV (blue circles). Curves: Best-fit reconstructed energy deposition profiles for ICS generated by 236 MeV electrons assuming a Gaussian spectrum (red dashed) and radiation model (red dotted) and 345 MeV electrons assuming a Gaussian spectrum (blue solid) and radiation model (blue dot-dashed). (**b**) Corresponding unfolded ICS spectra. Red and blue shading: reconstruction uncertainty. Inset: Electron spectra for the two shots.

Red and blue data points in Fig. 6a show background-subtracted energy deposition profiles of ICS generated on two separate shots, for which electron bunches had peak energy $236 \pm 14$ MeV ($\gamma _e = 462)$ and $345\pm 13$ MeV ($\gamma _e = 675$), respectively (see red dashed and blue solid curves in the inset of Fig. 6b). We achieved the lower and higher electron energies by tuning plasma density to $n_e = 4 \times 10^{18}$ cm$^{-3}$ and $6 \times 10^{18}$ cm$^{-3}$, respectively. Red dashed and blue solid curves in Fig. 6a show best-fit energy deposition profiles from the unfolding process; corresponding curves in the main panel of Fig. 6b show unfolded ICS spectra of the form of Eq. (7). Red and blue shading around both pairs of curves represents unfolding uncertainty. Table 4 lists ICS X-ray parameters $E_x$ and $\sigma _{E_x}$ of the unfolded spectra, along with corresponding electron parameters for each shot. The unfolded $E_x$ values for the two shots stand in the ratio $E_x^{(236)}/E_x^{(345)} = 0.63 \pm 0.13$, whereas the expected $\gamma _e^2$ scaling of $E_x$ would yield a ratio $0.47 \pm 0.09$, assuming identical laser frequency $\omega _0$ on both shots. While these ratios agree within the combined stated uncertainty, a possible reason for the discrepancy is that the laser pulse driving the denser plasma experienced a larger redshift, thus shifting the more energetic X-ray peak to lower energy.

**Table 4:** Electron parameters (left columns) and unfolded ICS X-ray parameters (right columns) based on a Gaussian model (Eq. 7), for two shots producing different peak energies $E_e$ and numbers $N_e$ of quasi-monoenergetic accelerated electrons.

	Electron parameters		Unfolded ICS parameters		
	$E_{pk}$ (MeV)	$N_{e}$ (>150 MeV)	$E_{x}$ (keV)	E spread ($\sigma _{E_x}$)	$N_{phot}$ (FWHM)
Shot 1	$236 \pm 14$	$1.5 \times 10^{9}$	$1040 \pm 90$	$410 \pm 50$	$8.2 \pm 2.0 \times 10^7$
Shot 2	$345 \pm 14$	$1.3 \times 10^{9}$	$1640 \pm 190$	$720 \pm 140$	$4.9 \pm 1.0 \times 10^7$

Table 4 presents the statistical average and standard deviation for $E_x$ and $\sigma _{E_x}$ for each shot. The two unfolded peaks are separated by more than their combined standard deviation and the unfolded value of $E_x$ for one peak falls outside of the FWHM of the second peak for 100% of trials. We estimate a resolution of the unfolded ICS peak energy $E_x$ to be $\sim 10\%$, determined primarily from uncertainty in the subtracted bremsstrahlung contribution in layers 10–20. The width of the spectrum, given only a lower bound as a physical constraint, has an uncertainty of $\sim 15\%$ and gives the approximate bandwidth limit of the unfolding procedure for narrowband sources in X-ray mixtures.

Simulations of the ICS spectrum require a good understanding of the 3-D laser intensity and the 6-D electron phase space to get accurate results of the farfield radiation spectrum^60^. However, the use of a plasma mirror makes it difficult to know the exact intensity and spectrum of the scattering laser pulse. Instead, a radiation model based on theory from Esarey et al.^28^ can be used to calculate the anticipated spectral shape, including harmonics, generated by the measured electron spectrum scattering from a laser pulse of central frequency $\omega _0$ and laser strength parameter $a_0$ (see Supplementary Material). The calculation integrates over observation angles that would contribute to signal in the stack and assumes the central frequency of the scattering laser can be redshifted by a percent of the original e.g. $\omega _{scatter} = RS \omega _0$ where $RS \le 1$. Calculations assuming several different values of $a_0$ in the range $0.1 \le a_0 \le 1.3$ were performed and the spectra resulting from other $a_0$ values within these bounds can be interpolated to provide a set of solutions to compare with the Gaussian model.

An unfolding based on this radiation model finds $RS = 0.65 \pm 0.1~(0.6 \pm 0.1)$ and $a_0=0.55 \pm 0.2~(0.48 \pm 0.12)$ to be the values that best fit the measured energy profile $D_i^{(meas)}$ ($1\le i \le 24$) for shot 1 (shot 2). Figure 6a shows the calculated energy deposition profiles $D_i^{(calc)}$ (dotted and dash-dotted curves) based on the best fit values of RS and $a_0$ for the radiation model. The corresponding spectra from the model are shown in Fig. 6b as dotted and dash-dotted curves with shading corresponding to the uncertainty of the unfolding. The goodness of fit defined by the fitness function $F(\bar{p})$ (see “Methods”) is $\sim 3 \times$ smaller for the radiation model that incorporates the electron spectrum compared with the Gaussian assumption. Moreover, the values of $a_0$ agree to within combined uncertainty with independent estimates of the laser intensity 1 mm after the exit of the accelerator^61^.

## Discussion

**Figure 7:**
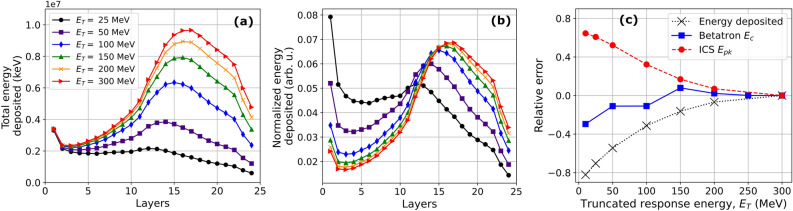
Effects of response matrix truncation. (**a**) Absolute and (**b**) normalized energy deposition profiles for bremsstrahlung X-rays with spectrum of the form of Eq. (5), with $E_0 = 300$ MeV, calculated using a response matrix that is truncated at $E_T =$ 25, 50, 100, 150, and 200 MeV, compared to one that includes the full 300 MeV range; (**c**) The relative error in the calculated total energy deposition (black dotted curve) and the unfolded parameters for betatron (blue solid curve) or ICS (red dashed curve) X-rays mixed with bremsstrahlung background: betatron critical energy $E_c$ (blue squares) and ICS peak energy $E_{pk}$ (red circles) are based on a response matrix truncated at energies given on the horizontal axis.
(ii) These truncation errors propagate into recovery of other X-ray spectra that are mixed with bremsstrahlung background, unless a reliable *independent* method for quantifying the shape and amplitude of the bremsstrahlung contribution is used. Our study of bremsstrahlung-only radiation (Fig. 4 and accompanying text), which was not available in prior work^42^, provided such a method by demonstrating the equivalence of bremsstrahlung spectra generated by unfolding a measured energy deposition profile with a spectrally complete response matrix, on the one hand, or generated by Geant4 simulations based on independently measured electron charge and spectrum, on the other.
  This equivalence gives us two methods for accurately unfolding mixed X-ray spectra, depending on whether the contributing sources are spectrally separated or overlapping. Our study of keV betatron radiation + bremsstrahlung from 25 μm-thick Kapton (Fig. 5 and accompanying text) approximates the former case. Betatron and bremsstrahlung photons deposit their energy in stack layers that are separated sufficiently that the two spectra can be unfolded jointly, with their relative amplitude as an additional unfolding parameter. The primary requirement for accuracy is a response matrix that is spectrally complete for the bremsstrahlung contribution. Figure 7c illustrates the errors that propagate into the unfolded betatron critical energy $E_c$ (blue squares) as a result of truncating the matrix below $E_0$, which is 300 MeV for the case shown. The error in the unfolded $E_c$ increases to $\sim 30\%$, with the unfolded $E_c$ getting smaller as the truncation energy decreases from 300 to 10 MeV, even though these truncation energies all exceed the energy of any betatron X-ray photons. This happens because the unfolding program adjusts betatron X-ray parameters to compensate errors in the bremsstrahlung background. We obtain a betatron spectrum equivalent to that obtained with a complete response matrix by a second method—first simulating the bremsstrahlung energy deposition profile in Geant4 from independently-measured electron charge and energy and scaling it to the measured energy deposition in layers 8 through 24, then unfolding the remaining betatron energy deposition profile by itself. This is a direct consequence of the equivalence discussed above.
  Our study of 0.5–2 MeV ICS + bremsstrahlung from a 25 μm-thick Kapton plasma mirror (Fig. 6 and accompanying text) best illustrates the case of overlapping spectral content. Here, direct joint unfolding with relative spectral amplitude as a free parameter does not converge consistently to a common ICS spectrum, even when a complete response matrix is used. This is because the algorithm is sensitive to small variations in initial guesses in this case. Hence we must rely on subtracting Geant4-simulated bremsstrahlung energy deposition profiles from the raw profiles, then unfolding background-subtracted profiles, the method used to obtain ICS spectra shown in Fig. 6. To illustrate the sensitivity of the unfolded ICS peak energy $E_{pk}$ to small variations in the bremsstrahlung spectrum, we started with the Geant4-simulated and scaled energy deposition profile for Shot 1 from the ICS X-rays section, then generated an ensemble of 6 additional bremsstrahlung profiles with different amplitudes based on the total energy deposited from increasingly truncated response matrices (see Fig. 7a). We then subtracted these bremsstrahlung profiles from the measured ICS energy deposition profile to generate an ensemble of 7 (including the original simulated profile) background-subtracted ICS profiles that were unfolded based on Eq. (7). The red circles in Fig 7c show the result of this exercise. As the truncation energy decreased to 10 MeV, the amplitude of the subtracted bremsstrahlung decreased by about $\sim 80\%$, resulting in $\sim 60\%$ larger unfolded ICS energies, a significantly larger effect than seen with the betatron + bremsstrahlung unfolding due to the overlapping spectral content.
  For the case of MeV betatron radiation studied by Jeon et al.^42^, the addition of a bremsstrahlung background from GeV electrons will have a more significant effect on the resulting unfolded parameters than we observe here for three reasons: (1) GeV electrons will generate more bremsstrahlung radiation than MeV electrons; (2) GeV photons will deposit significantly more energy in the stack than MeV photons; (3) A combination of a 200 μm-thick aluminum foil and 300 μm-thick LANEX screen is a significantly more efficient bremsstrahlung converter than 25 μm-thick Kapton. Our results indicate that the unfolded betatron results would not be reliable without a response matrix that extends to the bremsstrahlung cutoff energy (rather than truncated at 20 MeV) and a better independent quantification of the isolated bremsstrahlung contribution.
(iii) The assumed radiation models should be based on physical models wherever possible, especially when considering mixtures of X-rays. Results from solid target experiments often rely on two-temperature exponential or Boltzmann distributions for estimating the photon spectrum since the accelerated electron spectrum is broadband and difficult to measure^62^. However, LWFA electron bunches can be quasi-monoenergetic and measured with a high level of accuracy using magnetic spectrometers, requiring a better parameterization of each X-ray source. The standard for unfolding betatron radiation has been based on the synchrotron model (Eq. 1)^46^, but LWFA bremsstrahlung has been unfolded using the same models as in solid target experiments^42^. Figure 5b and Table 3 illustrate how the unfolded parameters from the mixture of betatron + bremsstrahlung X-rays can differ by $\sim 30\%$ simply from differences in the bremsstrahlung model in layers 1–4. Here we can conclude that the Kramers’ law bremsstrahlung model better fits the measured energy deposition because the fitness function (Eq. 9) is smaller. However, the difference in unfolded parameters from the two bremsstrahlung models is not significant for the tantalum bremsstrahlung X-rays, indicating that more complex models will not necessarily result in increased accuracy of the unfolded X-rays. The bremsstrahlung models used for the first time in our work incorporate the many temperature distribution and cutoff energy with higher accuracy than less physical models. Similarly, the ICS X-ray source is a case where a physical model that depends on knowledge of the independently measured electron spectra can provide a better fit to the measured energy deposition, however at a cost of unfolding time. For the ICS parameters presented here, the Gaussian assumption still falls within the uncertainty of the measured energy deposition and unfolds the critical photon parameters without requiring knowledge of the electron spectra. Nonlinear ICS in which $a_0$ exceeds 1 is just such a case where a more complex model may be necessary to unfold the harmonics of the fundamental, $4 \gamma _e^2 E_L$. Additionally, unfolding the spectra of X-rays radiated by electrons with multiply-peaked energy distributions will require models that incorporate such distributions explicitly.

Thorough understanding of each LWFA X-ray source is essential to unfolding the characteristic radiation parameters such as betatron critical energy, bremsstrahlung average energy and ICS peak energy accurately using a stack calorimeter. Generally this approach does not guarantee a unique solution for the incident photon spectrum. Nevertheless, past applications of stack calorimetry have diagnosed the spectra of simple X-ray sources, i.e. those consisting of one or two types of incident X-rays of broad spectral content, by simulating the detectors response to mono-energetic photons and feeding a guessed spectrum into a forward-fitting algorithm that minimizes the difference between measured and calculated signals^44–46,50^. Here, we have built on this success by first unfolding isolated (e.g. betatron- and bremsstrahlung-dominated) and combined X-ray sources with minimally overlapping spectra (e.g. betatron + bremsstrahlung from 25 μm-thick Kapton), for which uncertainty is minimal, then using these results as a basis for unfolding more complex X-ray mixtures with overlapping and narrowband spectra (e.g. ICS from a 25 μm-thick Kapton plasma mirror). These studies convey three key lessons for unfolding spectra accurately:

(i) The response matrix *must* cover the full range of photon energies that deposit energy in the stack. For a bremsstrahlung beam described by Eq. (5), for example, photons with energy beyond $E_{avg}$ represent $<25\%$ of total photons, yet they dominate the *shape* and *amplitude* of the deposition profile (see Figs. 4a and 5b, red $\times$’s and green circles). Figure 7a,b illustrate how truncating the response matrix at energies below the cutoff energy (here 300 MeV) affects the calculated absolute and normalized energy deposition profiles, respectively. Truncation at 200 MeV (Fig. 7a,b, yellow $\times$’s) already introduces $\sim 10\%$ error in total deposited energy (see also Fig. 7c, black $\times$, dotted curve) with minimal affect on the normalized shape. Errors in energy deposition grow to $\sim 30\%$ with truncation at 100 MeV [Fig. 7a,b, blue diamonds], and increase further with lower truncation energies, while also distorting the shape of the calculated energy deposition profile. Thus, to unfold LWFA bremsstrahlung spectra accurately, a spectrally complete response matrix, i.e. one extending to the maximum electron energy in the bunch, is essential.

Future direction for stack designs and implementation requires an improvement on the data collection and unfolding repetition rate. Currently, using a least squares optimization algorithm, each single parameter case (betatron and bremsstrahlung dominated) converge to the solutions presented here in $\sim 1$ s. The case of multiple parameters (bremsstrahlung + betatron and ICS dominated) converges in $\sim 10$ s on a lab grade laptop. These algorithms can easily be transferred to manycore processors since each unfolding is performed 100 times and each run is independent. These computations can be parallelized to reduce the unfolding time by a factor of 100 or more to $\le 100$ ms. To achieve data acquisition rates commensurate with such computational speed, image plates will need to be replaced with prompt scintillators compatible with $\sim$10 Hz LWFA repetition rates^63,64^. In this geometry, plastic scintillators or scintillating fiber arrays alternate with absorbing material and the side of the stack is imaged with a camera or can be connected directly to photomultiplier tubes (PMTs)^65^. The analysis to generate the measured energy deposition profile $D^{(meas)}$ can also be parallelized since the operations on image data are independent. Moreover, the transmission speed of data along Gigabit-ethernet or USB 3.0 cables is $\sim 5{-}10$ Gbps and can transfer typical image sizes of 5 Mb in $<10$ ms. Cameras can already operate at the necessary 100 fps for this application. The limiting factor on the speed of unfolding is most likely in the conversion of data to a format for computation on a manycore processor. In total, current technology would allow a prompt scintillator based stack to operate at a minimum of 0.1 to 2 Hz, providing a method for actively unfolding spectra during LWFA experiments where emitted radiation provides a metric for the acceleration process, e.g. enhanced betatron radiation from direct laser acceleration (DLA) or higher order harmonics in non-linear ICS.

We have presented a set of unfolded secondary X-ray spectra spanning over 4 orders of magnitude in energy from LWFA accelerated electrons with energies between 230 and 550 MeV. The LWFA and target geometry can be tuned to generate betatron, bremsstrahlung or ICS dominated sources as well as a regime in which both betatron and bremsstrahlung contribute to the stack. We present unfolding of betatron radiation with a critical energy of $14\pm 1.5$ keV and betatron radius of $1.0\pm 0.1~\upmu$m which are compared with independent measurements using a X-ray sensitive CCD and simulations from CLARA2. Bremsstrahlung from an 800 μm-thick tantalum target is unfolded with an average energy of $35\pm 4$ MeV and $4.2\pm 0.8\times 10^8$ photons within the FWHM and is compared with Geant4 simulations. Thin-target bremsstrahlung from 25 μm-thick Kapton includes contribution from both betatron and bremsstrahlung and the unfolded critical energy of the betatron source is $12\pm 3$ keV and the average bremsstrahlung energy is $15\pm 3$ MeV, spanning 3 orders of magnitude in a single shot. Finally, ICS dominated radiation from electron bunches with different peak energies was unfolded to observe a shift in peak ICS energy from $1060\pm 90$ keV to $1.64\pm 0.19$ MeV and a total of $8.2\pm 0.2\times 10^{7}$ and $4.9\pm 1.0\times 10^{7}$ photons in the FWHM, respectively. The ICS shots were compared with an electron dependent model that unfolded a value for $a_0$ of $0.55\pm 0.2$ and $0.48\pm 0.12$ and a relative redshift (RS = $\omega _L/\omega _0$) in the laser central frequency of $0.65\pm 0.1$ and $0.6\pm 0.1$. The stack calorimeter is less sensitive to background and has a higher signal to noise ratio for the energy ranges presented here than similar spectrometers that rely on a Compton converter^39,40^ or Ross filter pairs^35,36^. Furthermore, stack calorimeters are compact in size, making them ideal detectors for characterizing X-ray sources from a variety of laser systems.

## Methods

### Laser wakefield electron acceleration

Pulses of 30 fs duration, 800 nm center wavelength from the DRACO laser at Helmholtz-Zentrum Dresden-Rossendorf (HZDR)^66,67^ were focused to spot size 20 μm (FWHM) with typical energy 2 J onto the entrance plane of a 3-mm or 5-mm-long He gas jet doped with 1% Nitrogen. The laser pulse fully ionized the helium, creating plasma of electron density in the range $4< n_e < 6 \times 10^{18}$ cm$^{-3}$, and drove a LWFA in the self-truncated ionization-injection regime^66,68^. A magnetic electron spectrometer^67^ with its entrance plane at $z = 30$ cm downstream of the gas jet exit determined the electron energy distribution for each shot. The spectrometer records the dispersed electron beam using a Konica Minolta OG 400 scintillating screen that is converted to charge per unit energy per pixel^69^ (see Fig. 1b, left panel for an example from the 3 mm jet) using the methods described in Section IV. of Kurz et al.^69^. The absolute charge calibration uncertainty for our system is 19% and is shown with vertical error bars at the quasi-monoenergetic peak in presented electron spectra, however the relative uncertainty from shot-to-shot variations in charge are much smaller than this. Errors in electron energy measurement $>200$ MeV arise primarily from pointing and divergence fluctuations of LWFA electrons entering the magnetic spectrometer^11^ and is $\sim 2\%$ for electrons in the range of 200–350 MeV. Electrons with energy $E_e<200$ MeV are recorded near the spectrometer’s focal plane and have $<1\%$ uncertainty determined by the accuracy of the magnetic field measurement. The electron spectra presented here consist of a quasi-monoenergetic peak with central energy in the range $200< E_e < 350$ MeV (Lorentz factor $390< \gamma _e < 685$), energy spread $\sim$ 20 MeV (FWHM), rms divergence 2 mrad and charge in the range $200< Q < 300$ pC, which is responsible for most X-ray production, and a weak poly-energetic, low-energy background. The 2% error in electron energy is indicated as horizontal error bars at the peak or average electron energy for quasi-monoenergetic electron spectra. The 5-mm jet yielded electrons with energy up to 550 MeV, with a stronger poly-energetic background.

### X-ray spectral reconstruction

We write the integrated energy deposited in layer *i* of the calorimeter as a vector with components $D_i (i=1,2,...,24)$. We wish to reconstruct from this the spectrum $dN/d(\hbar \omega )$ of incident X-rays, which we discretize as a vector $dN_j/d(\hbar \omega )$ describing the number of photons in bin *j* of energy $\hbar \omega _j$ and width $d(\hbar \omega _j)$. A stack response matrix $R_{ij}$ describes the energy deposited in layer *i* by photon of energy $\hbar \omega _j$ and relates $D_i$ to $dN _j/d(\hbar \omega)$ via^49^:

8 $$\begin{aligned} D_i \approx \sum _{j=1}^{N} \frac{dN_j}{d(\hbar \omega )} R_{ij} ~d(\hbar \omega _j), \end{aligned}$$

where the sum is over the number of energy bins, *N*. Here, $N \approx 1600$, with $d(\hbar \omega _j) = 1$ keV for 5 keV $< \hbar \omega _j < 200$ keV, $d(\hbar \omega _j) = 20$ keV for 200 keV $< \hbar \omega _j < 10$ MeV, $d(\hbar \omega _j) = 250$ keV for 10 MeV $<\hbar \omega _j < 200$ MeV, $d(\hbar \omega _j) = 1$ MeV for 200 MeV $< \hbar \omega _j < 400$ MeV and $d(\hbar \omega _j) = 5$ MeV for 400 MeV $< \hbar \omega _j < 600$ MeV. We generate $R_{ij}$ by simulating energy deposition in the stack’s absorbers and IPs by mono-energetic photon beams of different $\hbar \omega _j$ using Geant4^70^. A reconstruction begins with an initial guess of $\frac{dN_j}{d(\hbar \omega )}(\hbar \omega _j,\bar{p})$, which here is constrained to take the form of a physics-based analytic function of $\hbar \omega _j$, typically including a small set $\bar{p}$ of fit parameters, describing betatron, ICS or bremsstrahlung radiation, or a combination of them. Specific functions used for each type of X-ray source are presented in the Results. Knowledge of the presence and location of PMs and converters, and other experimental parameters, is critical in choosing appropriate functions. The most accurate models take the measured electron spectrum (Fig. 1b) specifically into account. However, models that do not depend explicitly on the electron spectrum are also useful for rapid, albeit approximate, results. In either case, a forward calculation using Eq. (8) generates a first-generation $D^{(calc)}_i$, which is compared to the measured energy distribution $D^{(meas)}_i$. A fitness function

9 $$\begin{aligned} F(\bar{p}) = \sum _{i=1}^{n} (D^{(meas)}_i - D^{(calc)}_i)^2 = \sum _{i=1}^{n} \left( D^{(meas)}_i - \left[ \sum _{j=1}^{N} \frac{dN_j}{d(\hbar \omega )} R_{ij} ~d(\hbar \omega _j) \right] \right) ^2, \end{aligned}$$

i.e. the sum of squared residuals between the calculated and measured energy, then evaluates the goodness of fit where, *n* denotes the number of layers. In subsequent iterations, $\frac{dN_j}{d(\hbar \omega )}(\hbar \omega _j,\bar{p})$ is varied in an effort to minimize $F(\bar{p})$. Here, we unfold the spectral shape, not the absolute value, of $\frac{dN_j}{d(\hbar \omega )}(\hbar \omega _j,\bar{p})$, by fitting to the energy distribution $D_i$ normalized to total deposited energy $\sum _{i=1}^n D_i$. The overall scaling is reintroduced after the completed unfolding to account for the total energy in the beam (see Supplementary Material for stack calibration). As in solving any complex inverse problem with incomplete information, convergence of the iterative procedure and uniqueness of any best fit solution cannot be guaranteed. Thus thorough tests of the sensitivity of results to initial guesses, awareness of experimental conditions, liberal use of physical constraints on the form of solutions and accurate evaluation of error are essential to achieving reliable results.

### Analyzing stack data

For each IP layer in the stack the deposited energy is integrated within the FWHM of the incident beam to determine the measured energy distribution in the stack, $D_i^{(meas)}$ (plotted in Fig. 2a). The divergence of the incident photon beams is found by averaging the divergence in each layer over the relevant layers for each X-ray source. The betatron divergence is found using only layer 1, while the divergence of the bremsstrahlung and ICS sources is averaged over layers 5–18 to avoid an overestimation caused by betatron contributions or scattering in the high Z layers. Table 5 compiles the measured beam divergence for each presented case, the radius of integration for $D_i^{(meas)}$ and the resulting energy deposited. In the case of the betatron + bremsstrahlung X-rays from a 25 μm-thick Kapton target, the energy deposition profile $D_i^{(meas)}$ is integrated over a radius corresponding to the *bremsstrahlung* HWHM of $3.4 \pm 0.1$ for unfolding both sources. The unfolded betatron spectrum is then scaled to the energy integrated within a radius of 7 mrad corresponding to the betatron HWHM for direct comparison with the betatron dominated case.

**Table 5:** Compiled divergence, integration radius and integrated energy for each X-ray source presented in the text.

	Beam HWHM (mrad)	Integration radius (mrad)	Total energy deposited (keV)
Betatron	$7.7 \pm 0.5$	7.7	$2.8 \pm 0.6 \times 10^8$
800 μm Ta bremsstrahlung	$5.7 \pm 0.2$	5.7	$1.0 \pm 0.2 \times 10^{11}$
25 μm Kapton bremsstrahlung (betatron)	$3.4 \pm 0.1$ ($7\pm 2$)	3.4 (7)	$2.7 \pm 0.5 \times 10^8$
ICS shot 1	$4.5 \pm 0.2$	4.5	$1.8 \pm 0.3 \times 10^9$
ICS shot 2	$3.5 \pm 0.3$	3.5	$1.4 \pm 0.3 \times 10^9$

The energy deposited for the 25 μm-thick Kapton bremsstrahlung case is integrated within the HWHM of the bremsstrahlung beam and then scaled to the betatron beam HWHM (shown in parentheses) after unfolding.

### Error management

Uncertainty and error in measured energy deposition distribution $D^{(meas)}_i$ propagate into uncertainties and errors in recovered X-ray spectra $dN_j/d(\hbar \omega )$, and must therefore be carefully evaluated. Calibration of IP sensitivity and scanner introduce uncertainty of order $\pm 20\%$ into the absolute value of measured energy (see Supplementary Material). Variability of the fading rate of IP luminescence (typically $0.78\pm 0.03$ when scanned 10–15 min after exposure)^71^ introduces additional uncertainty. Fortunately, most of this uncertainty affects only overall energy deposited and absolute energy of the beam, not the *shape* of the energy deposition from which $dN_j/d(\hbar \omega )$ is unfolded. Nevertheless, layer-dependent errors arise when IPs with different ages, manufacturing and usage histories, and distributions of defects are mixed together in a stack. Repeated exposures of the same IP yield up to $\sim 5\%$ rms variation in recorded PSL^72^. Based on this measurement, we estimated $\sim 10\%$ rms variations within a stack, to take into account age and sensitivity difference among different IPs. Such variations introduce uncertainty into the normalized shape of the energy distribution, and hence into parameters of the unfolded spectrum. To take this into account, we randomly generate a normal distribution of synthetic energy profiles $D_i^{(syn)}$ with standard deviation of $10\%$ around the measured profile $D_i^{(meas)}$. This ensemble of synthetic energy profiles then becomes the target for unfolding. Each iteration uses one distribution from the ensemble as a target; the procedure is repeated $\sim ~$100 times using different $D_i^{(syn)}$ to obtain an equivalent ensemble average and standard deviation for the spectrum $dN_j/d(\hbar \omega )$, and for a given model’s parameter set $\bar{p}$.

## Supplementary Information

Supplementary Information.

## Data Availability

The data that support the plots within this article and other findings of this study are available from the corresponding author upon reasonable request.
