# Supplementary material for: Compact spectroscopy of keV to MeV X-rays from a laser wakefield accelerator
Source: Sci Rep. 2021 Jul 13;11:14368. doi: 10.1038/s41598-021-93689-5 (PMC8277848; doi:10.1038/s41598-021-93689-5)
Supplement: Supplementary file 1 — Supplementary Information. [file 41598_2021_93689_MOESM1_ESM.pdf]

# Compact Spectroscopy of keV to MeV X-rays from a Laser Wakefield Accelerator

**A. Hannasch[1], A. Laso Garcia[2], M. LaBerge[1,2], R. Zgadzaj[1], A. Köhler[2], J. P. Couperus Cabadağ[2], O. Zarini[2], T. Kurz[2], A. Ferrari[2], M. Molodtsova[2], L. Naumann[2], T. E. Cowan[2,3], U. Schramm[2,3], A. Irman[2], and M. C. Downer[1,*]**

[1]The University of Texas at Austin, Department of Physics, Austin, Texas 78712-1081, USA.
[2]The Helmholtz-Zentrum Dresden-Rossendorf, Institute for Radiation Physics, 01328 Dresden, Germany
[3]Technische Universität Dresden, 01069 Dresden, Germany
[*]downer@physics.utexas.edu

## Supplementary Material

## Stack composition, response and calibration

The stack design is composed of alternating absorbing materials of varying Z and thickness including PMMA, Aluminum, Brass and Steel as outlined in table S1. Fuji BAS-MS image plates (IP) are placed behind each absorbing layer to record the energy deposited by ionizing radiation. IPs have a large dynamic range and are sensitive to ionizing particles, recording the two-dimensional energy deposition of a radiation source either directly from the X-rays or from secondary particles such as electrons or positrons[1]. The response curves in Fig. S1 are generated by simulating mono-energetic, non-divergent photon beams interacting with the stack in Geant4 and recording the energy deposited per photon in each IP. The simulated IPs have a composition based on Rabhi *et al.* (2016)[2].

| Layer | Thickness | Material | Layer | Thickness | Material |
|---|---|---|---|---|---|
| 1 | 2 mm | PMMA | 13 | 2 mm | Brass |
| 2 | 2 mm | PMMA | 14 | 3 mm | Brass |
| 3 | 3 mm | PMMA | 15 | 3 mm | Brass |
| 4 | 3 mm | PMMA | 16 | 3 mm | Brass |
| 5 | 5 mm | PMMA | 17 | 3 mm | Brass |
| 6 | 5 mm | PMMA | 18 | 3 mm | Brass |
| 7 | 5 mm | PMMA | 19 | 3 mm | Steel |
| 8 | 3 mm | Aluminum | 20 | 3 mm | Steel |
| 9 | 3 mm | Aluminum | 21 | 3 mm | Steel |
| 10 | 4 mm | Aluminum | 22 | 4 mm | Steel |
| 11 | 4 mm | Aluminum | 23 | 13 mm | Steel |
| 12 | 2 mm | Brass | 24 | 10 mm | Steel |

**Supplementary Table S1.** List of stack absorbing materials and thicknesses. Alternating BAS-MS image plates are placed in plastic packets after each absorbing layer to record the energy deposition by the incoming radiation. The design remained consistent for all radiation sources presented here.

Calibration of the stack was performed using Cs137 and Co60 sources with activities of 9.25 GBq and 10 GBq with 10% tolerance, respectively. The sources are 4 mm x 4 mm cylindrical capsules encased in $\sim 1.2$ mm of stainless steel and are housed in a lead shielded box with mechanical lead doors and a 30° aperture. Fig. S2 shows the set up used for these measurements and includes a magnet to disperse low energy electrons and a Pb collimator with 1.2 cm diameter to isolate the characteristic X-rays. The stack calorimeter was placed after the Pb collimator for 1, 5, 10 and 20 minute exposures and then scanned $\sim 30$ minutes after the 20 minute exposure. The energy deposition profile is found by integrating the signal within a circle of 1 cm diameter for each exposure and subtracting the BG from a region between each exposure (see Fig. S2). A SpectroTRACER scintillator based spectrometer was also exposed to each source to provide a calibrated measurement of the spectrum and a BeO ceramic dosimeter measured the calibrated energy used to calculate the total flux of each source.

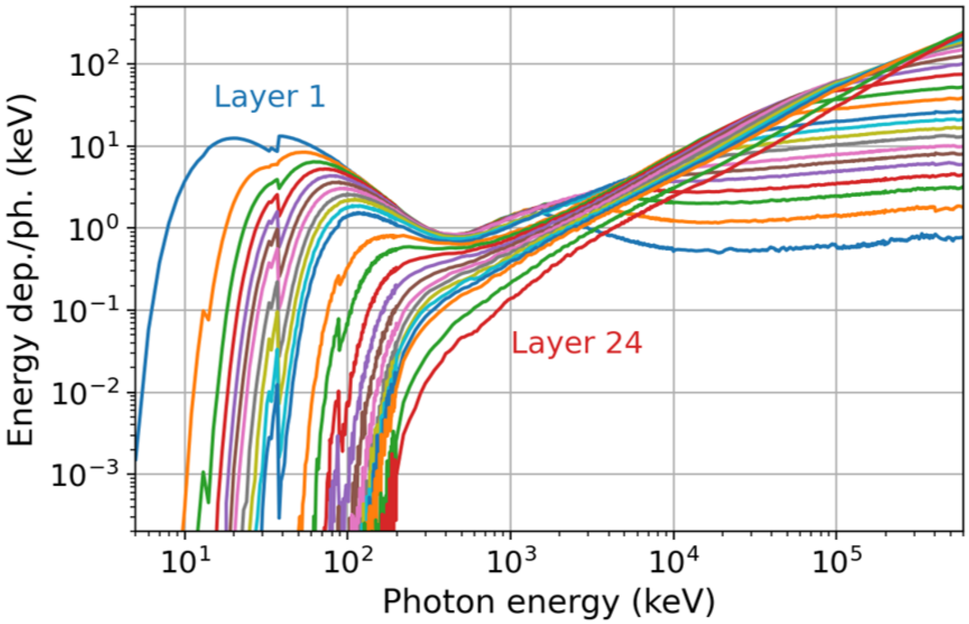


**Supplementary Figure S1.** Response curves in energy deposited per photon for layers 1 through 24 and from 5 keV to 600 MeV in varying step sizes.

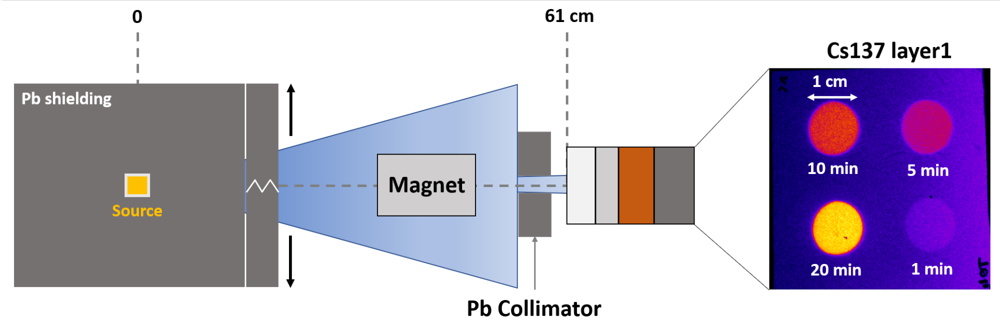


**Supplementary Figure S2.** Calibrated radioactive sources are housed in a Pb shielded box with a 30° aperture. Exposure times of 1, 5, 10 and 20 minutes are performed with the stack placed 61 cm away from the source capsule. A magnet and 1.2 cm Pb collimator are placed prior to the stack to remove secondary electrons and make multiple exposures possible.

## Response validation

A general unfolding of the Cs137 and Co60 source spectra is *not* the goal for this manuscript. We aim only to provide validation that the response matrix is accurate given the incident photon spectrum is known or well-understood. The bottom panels in Fig. S3(a) and (b) show the calibrated SpectroTRACER spectra (red dashed curve) for Cs137 and Co60, respectively. The characteristic energies are clearly visible in each case at 662 keV for Cs137 and at 1.17 and 1.33 MeV for Co60. Other visible features include a fluorescence peak at $\sim$ 88 keV from the Pb collimator as well as a Compton continuum resulting from characteristic X-rays scattering within the scintillator and then escaping. The scattered electrons in these events deposit the transferred energy in the scintillator and generate the additional background observed in the SpectroTRACER output. These Compton scattering events similarly occur within the absorbing materials in the stack and deposit energy in the image plates. However, energy deposition by Compton electrons or $e^+e^-$ pairs produced during the interaction of an X-ray with the absorbing material in the stack is calculated in the Geant4 Electromagnetic process and model classes when simulating the response matrix. Thus, the only source that could generate the measured energy deposition (see Fig. S3, black squares) would be from the characteristic X-rays and the Pb fluorescence peak. The Pb fluorescence peak amplitude can be estimated from the SpectroTRACER spectra and subsequently unfolded, while keeping the location of the peak and characteristic X-rays locked in place, to optimize the fit (see bottom panels of Fig. S3, blue solid curve).

Additional background from electron decay products generating bremsstrahlung through the steel capsule enclosing the sources can contribute to the stack profile as well. Simulations indicate that the amplitude of the background is lower by a factor $\sim 2-5\times10^{-4}$ compared with the characteristic peaks due to re-absorption in the source and surrounding steel capsule and does not contribute significantly to energy deposition in the stack. Fig. S3 (top panels) show that the agreement between the measured energy deposition profile (data points) and calculated energy deposition profile from the *assumed* spectrum including only the characteristic and Pb fluorescence peaks (blue solid curve) is within $\pm10\%$. These examples indicate that the response of the stack to photons in the range of 0.1 - 1.3 MeV is accurate to within 10%.

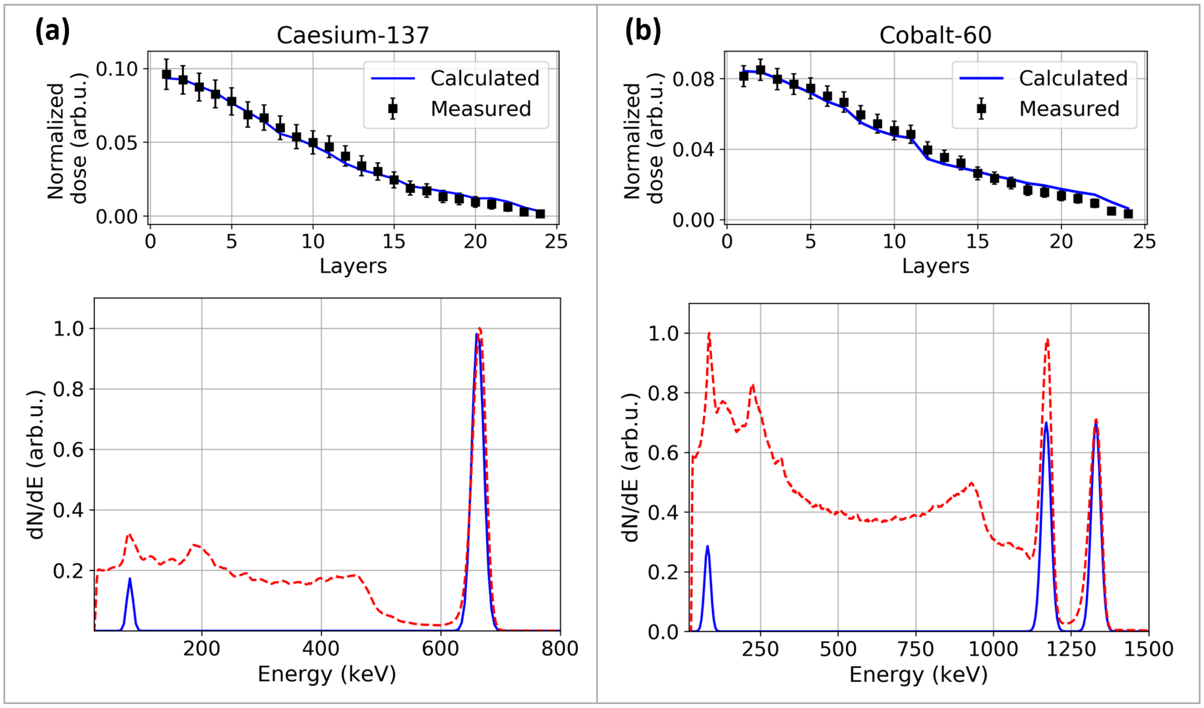


**Supplementary Figure S3.** Top panels compare the 20 minute exposure after background subtraction (data points) and calculated energy profiles (blue) for (a) Cs137 and (b) Co60. The corresponding incident photon spectrum without the Compton continuum (blue solid) and with the Compton continuum from the SpectroTRACER spectrometer (red dashed) are compared in the bottom panels.

### Total flux

A BeO detector[3] was used to measure the calibrated dose for each source in combination with a magnet and Pb collimator. The flux was calculated assuming only photons at the characteristic energy for Cs137 and Co60 and is $1.6\times10^5$ and $3.7\times10^5$ $cm^{-2}s^{-1}$ respectively. The total energy flux into the stack is also calculated assuming only photons at the characteristic energies and allows us to determine the scaling factor, $\alpha$, between PSL and energy for the image plates and scanner used in this experiment. This factor varies only mildly between the two sources, $\alpha_{Cs137} = 2.7\pm0.6\times10^{-4}$ PSL/keV and $\alpha_{Co60} = 3.0\pm0.5\times10^{-4}$ PSL/keV, and each are within error of the other. These values are smaller than similar calibrations performed in the field[1,4] but this can be caused by a lower sensitivity of the scanner used for our experiments and a lack of recent re-calibration[5]. For results presented here, we use $\alpha = 2.9\pm0.6\times10^{-4}$ PSL/keV to convert the PSL to energy deposition in the stack.

## Raw image plate data

Figure S4 provides the raw data for the full stack (24 layers) for all cases presented in the Fig. 2 of the main text and includes the second ICS shot from electrons with peak energy $345\pm14$ MeV. Raw scanner output was converted to PSL and cropped to the full IP size of $5\times5\,cm^2$ and an acceptance angle of 32 mrad. Each image plate is labeled with the layer and no other modifications, translations or rotations were performed beyond applying a "Fire" color map in ImageJ. The maximum pixel value is provided by the color bars at the bottom and was chosen to best illustrate the full stack information for each case.

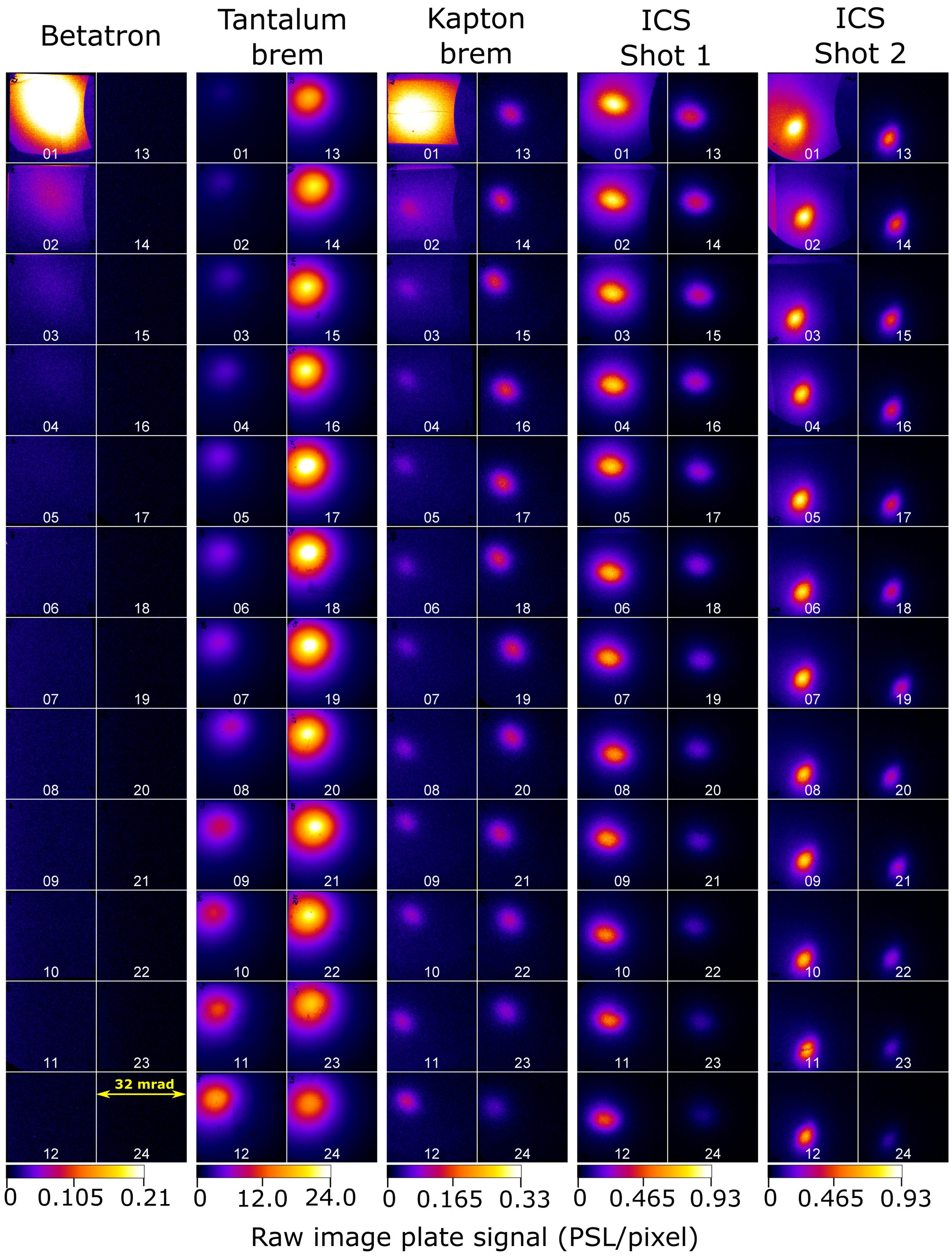


**Supplementary Figure S4.** Raw image plate data for all 24 layers in the stack and each X-ray source studied in the main text. Each layer is labeled and the PSL/pixel values are provided by the color bars below each set of raw data.

## CLARA2 simulations for betatron radiation

CLARA is a **CLA**ssical **RA**diation code that calculates radiation spectra, $d^2I/d\omega d\Omega$, in the farfield from the Liénard-Wiechert potentials[6].

$$\frac{d^2I}{d\omega d\Omega} = \frac{q^2}{16\pi\varepsilon_0 c}\left|\int_{-\infty}^{+\infty}\frac{\bar{n}\times\left[\left(\bar{n}-\bar{\beta}\right)\times\dot{\bar{\beta}}\right]}{(1-\bar{\beta}\cdot\bar{n})}\cdot e^{i\omega(t-\bar{n}\cdot\bar{r}(t)/c)}\right|^2 \tag{1}$$

The calculation takes the particle trajectories, $\bar{r}(t)$, and energy as inputs and is parallelized to run on large CPU clusters. To calculate the spectra from betatron emission the electron trajectories are found using a linear energy gain acceleration model where the electrons experience sinusoidal oscillations with a time-dependent amplitude, $r_b(t)$, and frequency, $\omega_b(t)$.

$$x(t) = r_b(t)\cos(\phi_b(t)) \tag{2}$$

Here, $x(t)$ is the 1-D particle path where

$$r_b(t) = r_{b0}\left(\frac{\gamma(t)}{\gamma_0}\right)^{-1/4} \text{ and } \phi_b(t) = \int_0^t \omega_b(t)dt \tag{3}$$

Both the oscillation amplitude and frequency depend on the time-dependent energy where $\omega_b(t) = \omega_p/\sqrt{2\gamma(t)}$. To calculate the single electron trajectory, the energy gain is estimated to be linear[7]

$$\gamma(t) = \gamma_0 + \frac{eE_z}{m_e c}t \tag{4}$$

where $E_z$ is the accelerating field and $\gamma_0$ is related to the phase velocity of the bubble upon injection such that $\gamma_0 \approx \gamma_\phi = 1/\sqrt{1-(v_\phi/c)^2} \approx \omega_0/\sqrt{3}\omega_p$[8,9]. For the experimental shots presented here, $n_e = 5\times10^{18}\ cm^{-3}$ and $\gamma_0 \sim 10$. The final energy $\gamma_f$ is then extracted from the measured electron spectrum from the experiment.

To find the betatron radius for an electron bunch with an arbitrary distribution of $r_{b0}$ values, a set of electron trajectories is calculated using equations (2)-(4) and input into CLARA2 to find the $r_{b0}$ dependent betatron spectra. The final betatron spectrum from the electron bunch can be found by weighting the set of CLARA2 spectra with the initial $r_{b0}$ distribution.

## Bremsstrahlung model approximations

Calculations of the cross-section based on the Born approximation hold for cases when $Z/137 < v/c$ and applies to the scattering of relativistic electrons presented here[10]. The differential cross-section in photon energy $\hbar\omega$ (neglecting screening effects) from an electron of energy $E_0$ is

$$\left(\frac{d\sigma}{d(\hbar\omega)}\right)_{Born} = \frac{16}{3}\frac{Z^2r_e^2\alpha}{\hbar\omega}\left(1-\frac{\hbar\omega}{E_0}+\frac{3\hbar^2\omega^2}{4E_0^2}\right)\left[\ln\left(\frac{2E_0(E_0-\hbar\omega)}{m_ec^2\hbar\omega}\right)-\frac{1}{2}\right] \tag{5}$$

Here, Z is the charge of the scattering nucleus, $\alpha$ is the fine structure constant and $r_e$ is the classical electron radius. For a high Z target and relativistic electrons ($E_0 >> 137mc^2Z^{-1/3}$), screening of the nucleus by atomic electrons can have an energy dependent effect on the cross-section and a correction to equation (5) is required. The cross-section when screening is considered can be written analytically as

$$\left(\frac{d\sigma}{d(\hbar\omega)}\right)_{screening} = \frac{16}{3}\frac{Z^2r_e^2\alpha}{\hbar\omega}\left[\left(1-\frac{\hbar\omega}{E_0}+\frac{3\hbar^2\omega^2}{4E_0^2}\right)\ln\left(183Z^{-1/3}\right)+\frac{1}{9}\left(1-\frac{\hbar\omega}{E_0}\right)\right] \tag{6}$$

For the screened case, the last term in the brackets contributes $\sim 3\%$ compared to the first term for tantalum ($Z = 73$) and can be dropped for the approximation without affecting the relative shape of the spectrum. Then, the photons emitted per energy bin can be estimated by integrating the cross-section over the electron energy loss and by assuming that the electron loses energy to radiation at a rate proportional to its energy, $dE/dx = -E/L_0$[10].

$$\frac{dN}{d(\hbar\omega)} = nN_e \int_{\hbar\omega}^{E_i} \frac{d\sigma}{d(\hbar\omega)} \frac{dE_0}{(-dE_0/dx)} = nN_eL_0 \int_{\hbar\omega}^{E_i} \frac{1}{E_0} \frac{d\sigma}{d(\hbar\omega)} dE_0$$

Here, $n$ is the target density, $L_0$ is the radiation length, $N_e$ the number of electrons and $E_i$ is the initial electron energy. Below a critical energy of $E_0 = 1600mc^2/Z$ the energy loss is dominated by collisions and the electron no longer contributes significantly to the photon spectrum. For tantalum, this energy is $\sim$ 11 MeV and would require that the highest energy electrons lose $\sim$ 98% of their energy. This energy loss translates to a tantalum thickness of $4\times$ the radiation length, or 1.2 cm. We performed the bremsstrahlung experiments with 800 $\mu m$ tantalum target thickness and so we should not lose more than 20% of the electron energy to radiation. This still requires the integration over the cross-section and the result is a piece-wise function to account for photon energies above and below the final electron energy.

$$\frac{dN}{d(\hbar\omega)} = nN_eL_0 \int_{\hbar\omega}^{E_i} \frac{1}{E_0} \frac{d\sigma}{d(\hbar\omega)} dE_0, \qquad E_f \leq \hbar\omega \leq E_i \tag{7a}$$

$$\frac{dN}{d(\hbar\omega)} = nN_eL_0 \int_{E_f}^{E_i} \frac{1}{E_0} \frac{d\sigma}{d(\hbar\omega)} dE_0, \qquad \hbar\omega < E_f \leq E_i \tag{7b}$$

The final electron energy $E_f$ is calculated based on the target thickness $t$ and radiation length $L_0$ of the material as $E_f = E_0 \exp(-t/L_0)$, where $E_0$ is the initial electron energy. This integration can be done analytically if we assume the form of the cross-section for full screening and remains computationally efficient since the only input is the initial electron energy and target material information. The photon spectrum can be written analytically as

$$\left(\frac{dN}{d(\hbar\omega)}\right)_{low} = \frac{C}{\hbar\omega}\left(\ln\frac{E_0}{E_f} + \hbar\omega\left(\frac{1}{E_0} - \frac{1}{E_f}\right) - \frac{3}{8}(\hbar\omega)^2\left(\frac{1}{E_0^2} - \frac{1}{E_f^2}\right)\right), \qquad \hbar\omega < E_f \leq E_0 \tag{8a}$$

$$\left(\frac{dN}{d(\hbar\omega)}\right)_{high} = \frac{C}{\hbar\omega}\left(\ln\frac{E_0}{\hbar\omega} + \hbar\omega\left(\frac{1}{E_0} - \frac{1}{\hbar\omega}\right) - \frac{3}{8}(\hbar\omega)^2\left(\frac{1}{E_0^2} - \frac{1}{(\hbar\omega)^2}\right)\right), \qquad E_f \leq \hbar\omega \leq E_0 \tag{8b}$$

where $C = 16Z^2r_e^2\alpha nN_eL_0/3$.

## ICS model calculations

An analytic expression for the energy radiated per unit frequency per unit solid angle by a single electron with Lorentz factor $\gamma_0$, oscillating in a linearly polarized plane wave laser pulse with strength parameter $a_0$ and central frequency $\omega_0$ is derived by Esarey *et al.* (1993)[11]:

$$\frac{d^2I}{d(\hbar\omega)d\Omega} = \sum_{n=1}^{\infty} \frac{e^2N_0^2}{16\pi\hbar\varepsilon_0 c}\left(\frac{k}{k_0}\right)^2 Res(k, nk_0) \times F_n \tag{9}$$

Here, $N_0$ is the number of laser periods, $n$ the harmonic number, $k_0$ the scattering wave number of the laser and $k$ is the emitted wave number of the radiation. The radiation for a given harmonic is peaked at a resonant frequency defined by the resonant function, $Res(k, nk_0)$:

$$Res(k, nk_0) = \left[\frac{\sin\bar{k}L/2}{\bar{k}L/2}\right]^2 \tag{10}$$

where $\bar{k} = nk_0 - k(1 + a_0^2/2 + \gamma_0^2\theta^2)/4\gamma_0^2$, $L$ is the length of the laser pulse $c\tau$ and $\theta$ is the observation angle with respect to the axis. This results in a resonant frequency of

$$\omega_n = \frac{4\gamma_0^2\omega_0 n}{1 + a_0^2/2 + \gamma_0^2\theta^2} \tag{11}$$

The relative width of the resonant function is $\Delta\omega/\omega_n = 1/nN_0$ and for a 800 nm, 30 fs pulse $N_0 = c\tau/\lambda_0 \approx 10$, contributing about 10% of the relative energy spread for the fundamental, $n = 1$.

The $F_n$ component of equation (9) is a harmonic amplitude function which gives the relative weight of a harmonic as a function of $a_0$, $\theta$ and $\gamma_0$[11]. For $a_0 << 1$ the only significant component is the fundamental, but as early as $a_0 \sim 0.3$ the second harmonic can play a significant role when integrating over observation angles. To account for electron energy spread and divergence, we integrate the single electron energy over the electron's phase space $N_e f(\gamma, \theta_e)$, where $\theta_e$ is the angle the electron travels with respect to the axis and $f(\gamma, \theta_e)$ is normalized to the total electron number $N_e$:

$$\frac{dI_{tot}}{d(\hbar\omega)} = 2\pi \int_0^{\theta_{max}} \sin\theta d\theta \int d\theta_e \int f(\gamma, \theta_e) \frac{d^2 I}{d(\hbar\omega) d\Omega}(\theta - \theta_e, \gamma) d\gamma. \tag{12}$$

The integration over $\theta_e$ and $\gamma$ can be limited to the extent of the beam, and the maximum observation angle, $\theta_{max}$ is chosen as the acceptance angle for the stack measurements. Electrons that travel at an angle $\theta \neq 0$ with respect to the axis will still emit radiation on axis that is redshifted according to equation (11). The inclusion of $\theta_e$ simply acts to average the angular distribution at *any* observation angle. This effect is illustrated in Fig. S5(a). The total energy radiated from an electron bunch with a peak energy of 250 MeV ($\gamma_0 = 490$) and $\sigma_\gamma/\gamma_0 = 0.065$ is calculated assuming a non-divergent electron bunch (red curves) and an electron bunch with 2-D gaussian profile and $\sigma_e = 1.7$ mrad (black curves). The electron divergence and energy spread were chosen to closely match the measured bunch parameters. The distribution is integrated up to a normalized angle $\gamma\theta$ of 0.2 (solid), 0.3 (dashed), 0.6 (dot-dashed) and 1 (dotted) to clearly illustrate the effect of the electron divergence. The non-divergent electron case (red curves) illustrate the redshifting of the spectrum that occurs at off-axis observation angles, starting at $\omega_x \approx 4\gamma_0^2\omega_0$ and reducing by about 10% after integrating over $\gamma\theta = 1$. The divergent electron case (black curves) maintain the same peak location and width for all final integration values and only increases in amplitude as more electrons are included in the summation. The resulting shape is the same as that reached from a non-divergent electron bunch at $\gamma\theta = 1$. By dropping the additional integral over $\theta_e$ the final spectrum remains valid for a divergent electron bunch as long as the integration over observation angles extends to $\gamma\theta = \gamma\theta_e$.

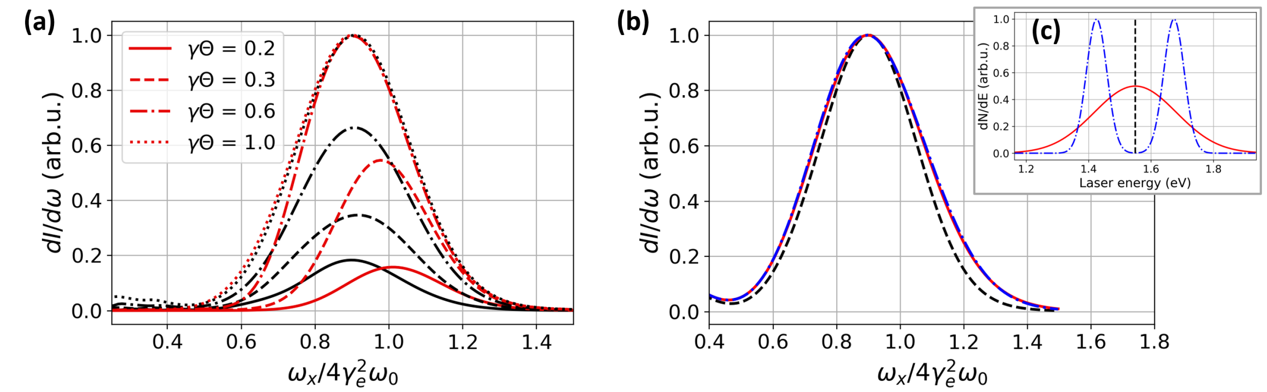


**Supplementary Figure S5.** (a) The calculated ICS spectra for $a_0 = 0.01$ and laser frequency $\omega_0$ from an electron bunch with 6.5% energy spread and no divergence (red curves) and from a bunch with divergence $\sigma_e = 1.7$ mrad (black curves). Each curve is the result of integrating Eq (12) up to $\gamma_0\theta_{max}$ of 0.2 (solid), 0.3 (dashed), 0.6 (dot-dashed) and 1.0 (dotted). (b) The calculated ICS spectrum from non-divergent electrons integrated up to $\gamma_0\theta_{max} = 1$ from a single laser frequency of $\hbar\omega_0$ =1.55 eV (black dashed), a Gaussian laser spectrum with 8.5% energy spread (red solid) and from a laser spectrum with two peaks separated by 9% (blue dot-dashed). The laser spectra that correspond to the calculations in (b) are shown in (c).

After dropping the integration over $\theta_e$ we can write the total energy radiated from an electron bunch per unit $\hbar\omega$ as

$$\left\langle \frac{dI_{tot}}{d(\hbar\omega)} \right\rangle_\phi = 2\pi\alpha N_e N_0^2 a_0^2 \int_0^{\theta_{max}} \sin\theta d\theta \int d\gamma f(\gamma)\gamma^2 \left( \frac{1+\gamma^4\theta^4}{(1+\gamma^2\theta^2)^4} \right) Res(k, nk_0) \tag{13}$$

where we have assumed $a_0 \ll 1$, $\theta \ll 1$ and averaged over the azimuthal angle $\phi$.

Another factor that slows down computation is the inclusion of a realistic scattering laser spectrum. To implement this feature, an integral over the laser spectrum needs to be added. Additionally, the central laser wavelength is expected to redshift

as the laser pulse depletes and energy is transferred to the wake. The depletion length for these accelerators is[8]

$$L_{pd} \simeq \frac{\omega_0^2}{\omega_p^2} c\tau = \frac{n_c}{n_e} c\tau \tag{14}$$

where $n_c$ is the critical density and $c\tau$ is the spatial extent of the laser pulse. The redshift can be estimated from the depletion length as

$$\frac{\Delta\omega}{\omega_0} \sim \frac{L_{acc}}{L_{pd}} \tag{15}$$

For conditions in the experiment, $n_c = 1.7 \times 10^{21}$ cm$^{-3}$ and $n_e = 4 \times 10^{18}$ cm$^{-3}$ and $n_e = 6 \times 10^{18}$ cm$^{-3}$ for shot 1 and 2 in the ICS section of the main text, respectively. The acceleration length in the STII regime is typically on the order of 1 mm and the depletion length is between 4 and 3 mm respectively resulting in a shift of 25% and 40%. Moreover, the nominal energy spread of the laser is $\sigma_L/E_L$ is $\sim 0.03$ and the broadening from the LWFA is expected to be primarily caused by the laser depletion and redshift. Structures within the laser spectrum do not impart the same features on the resulting ICS spectrum as long as the spread remains less than the relative broadening caused by the electron divergence and energy spread as well as the angular integration. Fig. S5(b) shows the calculated ICS spectra integrated over $\theta\gamma = 1$ from an electron bunch ($\sigma_\gamma/\gamma_0 = 0.065$) scattering from the three different laser spectra shown in Fig. S5(c). The nominal laser assumes a single laser frequency $E_{L0} = \hbar\omega_0 = 1.55$ eV (black dotted) and compares it with a Gaussian spectrum centered at $E_{L0} = 1.55$ eV and 8.5% spread (red solid) and a spectrum with two peaks placed at $E_{L0} \pm 0.9 E_{L0}$ and 2% spread (blue dot-dashed). The cases assuming $\sim 9\%$ laser spread result in the same calculated ICS spectrum regardless of the relative shape of the spectrum. Furthermore, they add only $\sim 2\%$ to the total energy spread making the central frequency the primary feature of the laser pulse to include in the calculation. Thus, for linear ICS where $a_0 \ll 1$, the energy spectrum can be approximated as a Gaussian function with mean photon energy $E_x$ and variance $\sigma_{E_x}$.

## References


1. Bonnet, T. *et al.* Response functions of imaging plates to photons, electrons and 4He particles. *Rev. Sci. Instruments* **84**, 103510, DOI: 10.1063/1.4826084 (2013).
2. Rabhi, N. *et al.* Calibration of imaging plates to electrons between 40 and 180 MeV. *Rev. Sci. Instruments* **87**, 053306, DOI: 10.1063/1.4950860 (2016).
3. Jahn, A., Sommer, M., Ullrich, W., Wickert, M. & Henniger, J. The BeOmax system - Dosimetry using OSL of BeO for several applications. *Radiat. Meas.* **56**, 324–327, DOI: 10.1016/j.radmeas.2013.01.069 (2013).
4. Boutoux, G. *et al.* Study of imaging plate detector sensitivity to 5-18 MeV electrons. *Rev. Sci. Instruments* **86**, 113304, DOI: 10.1063/1.4936141 (2015).
5. Zeil, K. *et al.* Absolute response of Fuji imaging plate detectors to picosecond-electron bunches. *Rev. Sci. Instruments* **81**, 013307, DOI: 10.1063/1.3284524 (2010).
6. Pausch, R. *et al.* How to test and verify radiation diagnostics simulations within particle-in-cell frameworks. *Nucl. Instruments Methods Phys. Res. Sect. A: Accel. Spectrometers, Detect. Assoc. Equip.* **740**, 250–256, DOI: 10.1016/j.nima.2013.10.073 (2014).
7. Glinec, Y. *et al.* Direct observation of betatron oscillations in a laser-plasma electron accelerator. *EPL* **81**, 1–4, DOI: 10.1209/0295-5075/81/64001 (2008).
8. Lu, W. *et al.* Generating multi-GeV electron bunches using single stage laser wakefield acceleration in a 3D nonlinear regime. *Phys. Rev. Special Top. - Accel. Beams* **10**, 1–12, DOI: 10.1103/PhysRevSTAB.10.061301 (2007).
9. Albert, F. *et al.* Angular dependence of betatron X-ray spectra from a laser-wakefield accelerator. *Phys. Rev. Lett.* **111**, 1–5, DOI: 10.1103/PhysRevLett.111.235004 (2013).
10. Bethe, H. & Heitler, W. On the Stopping of Fast Particles and on the Creation of Positive Electrons. *Proceeding Royal Soc. A* **146**, 83–112, DOI: 10.1142/9789812795755{_}0006 (1934).
11. Esarey, E., Ride, S. & Sprangle, P. Nonlinear Thomson scattering of intense laser pulses from beams and plasmas. *Phys. Rev. E* **48**, 3003–3021, DOI: 10.1103/PhysRevE.48.3003 (1993).
